# Dissociable influences of reward motivation and positive emotion on cognitive control

**DOI:** 10.3758/s13415-014-0280-0

**Published:** 2014-04-15

**Authors:** Kimberly S. Chiew, Todd S. Braver

**Affiliations:** 1 Center for Cognitive Neuroscience, Duke University, Durham, NC USA; 2 Department of Psychology, Washington University in St. Louis, St. Louis, MO USA; 3 Center for Cognitive Neuroscience, Duke University, Levine Science Research Center, Box 90999, Durham, NC 27708 USA

**Keywords:** Emotion, Motivation, Incentive, Positive affect, Cognitive control

## Abstract

It is becoming increasingly appreciated that affective and/or motivational influences contribute strongly to goal-oriented cognition and behavior. An unresolved question is whether emotional manipulations (i.e., direct induction of affectively valenced subjective experience) and motivational manipulations (e.g., delivery of performance-contingent rewards and punishments) have similar or distinct effects on cognitive control. Prior work has suggested that reward motivation can reliably enhance a proactive mode of cognitive control, whereas other evidence is suggestive that positive emotion improves cognitive flexibility, but reduces proactive control. However, a limitation of the prior research is that reward motivation and positive emotion have largely been studied independently. Here, we directly compared the effects of positive emotion and reward motivation on cognitive control with a tightly matched, within-subjects design, using the AX-continuous performance task paradigm, which allows for relative measurement of proactive versus reactive cognitive control. High-resolution pupillometry was employed as a secondary measure of cognitive dynamics during task performance. Robust increases in behavioral and pupillometric indices of proactive control were observed with reward motivation. The effects of positive emotion were much weaker, but if anything, also reflected enhancement of proactive control, a pattern that diverges from some prior findings. These results indicate that reward motivation has robust influences on cognitive control, while also highlighting the complexity and heterogeneity of positive-emotion effects. The findings are discussed in terms of potential neurobiological mechanisms.

**Electronic supplementary material:**

The online version of this article (doi:10.3758/s13415-014-0280-0) contains supplementary material, which is available to authorized users.

In navigating the rich and complex environment of daily life, emotional and motivational influences on information are critical to the process of selecting and implementing goal-directed behavior. At its most fundamental level, much of goal pursuit revolves around pursuing outcomes that are pleasurable or adaptive, and avoiding outcomes that are not. Consequently, growing interest has been directed toward elucidating the mechanisms by which emotional and motivational significance influence cognitive control and goal-directed behavior. Prior experimental research has suggested influences on cognitive control arising from both emotional manipulations, in which valenced subjective experience is directly induced (e.g., with mood inductions or emotional stimuli; Dreisbach, 2006; Dreisbach & Goschke, 2004; Fredrickson & Branigan, 2005; Gray, 2001; Isen & Daubman, 1984; Isen, Daubman, & Nowicki, 1987; Rowe, Hirsh, & Anderson, 2007; van Wouwe, Band, & Ridderinkhof, 2011), and from motivational manipulations, in which motivational state is altered with rewarding or punishing incentives (Chiew & Braver, 2013; Dambacher, Hübner, & Schlösser, 2011; Engelmann, Damaraju, Padmala, & Pessoa, 2009; Jimura, Locke, & Braver, 2010; Locke & Braver, 2008; Padmala & Pessoa, 2011). Yet for the most part, these effects have been studied in independent investigations, without direct comparisons. Theoretical and experimental literature suggests that emotion and motivation are highly interrelated but distinct concepts (Carver, 2003; Lang & Bradley, 2008; Roseman, 2008), but the extent to which their influences on human cognition may be considered dissociable remains an open question. Moreover, two recent reviews (Chiew & Braver, 2011; Dreisbach & Fischer, 2012) have suggested that emotion and motivation—specifically, focusing on positive affect and reward incentive—may influence cognitive control in distinct ways. To address outstanding questions from previous literature, the present study aims to clarify the relationship between positive emotion and reward motivation constructs and their influences on cognition through direct empirical comparison.

Common versus distinct influences of emotion and motivation on cognitive control can potentially be productively viewed in terms of the dual mechanisms of control (DMC) framework (Braver, Gray, & Burgess, 2007). The DMC framework postulates that cognitive control can be understood as operating via two primary modes: proactive and reactive. Proactive control is thought to provide relatively tonic maintenance of goal information, whereas reactive control is thought to act as a flexible form of “late correction” in response to performance monitoring. These modes can be characterized by changes in cognitive performance and temporal control dynamics.

Consistent with the role of reward motivation as a critical determinant of goal selection and maintenance, motivational manipulations (i.e., reward incentives) have been found to enhance cognitive maintenance and proactive control. Given evidence from primates that task-related reward value modulates dopaminergic (DA) input to the prefrontal cortex (PFC; Leon & Shadlen, 1999; Watanabe, 1996) as well as evidence that DA mechanisms support working memory and cognitive control activity in PFC (Arnsten, Cai, Murphy, & Goldman-Rakic, 1994; Sawaguchi & Goldman-Rakic, 1991, 1994), it appears that enhanced DA input to the PFC may be a key neural mechanism underlying motivation–cognitive control interactions. Findings from neuroimaging studies in humans provide additional support for this account, indicating that motivational incentives enhance proactive control and brain activity in the PFC and reward-related regions (Locke & Braver, 2008; Padmala & Pessoa, 2011; Pochon et al., 2002). Several of these findings have been demonstrated using the AX-continuous performance task (AX-CPT; Cohen, Braver, & O’Reilly, 1996; Servan-Schreiber, Cohen, & Steingard, 1996), which provides relative indices of proactive and reactive cognitive control.

On each trial, the AX-CPT requires participants to respond to a cue–probe pair (e.g., letters presented sequentially). One specific combination requires a target response (i.e., the letter “A” followed by the letter “X”; AX trial) with all other cue–probe combinations requiring a nontarget response. Target AX trials occur with a high frequency (typically 70%), leading to interference for two low-frequency cue–probe pairs (10% each): AY (target cue, nontarget probe) and BX (nontarget cue, target probe). BY (nontarget cue, nontarget probe) trials also occur as a low-frequency (10%) control condition. In AY trials, interference arises from cue-related expectancy, and can be used as an index of proactive control. In contrast, in BX trials interference arises via response bias to the probe, and can be used as an index of reactive control. AY and BX trial performance indices tend to be antagonistic (e.g., Braver, Satpute, Rush, Racine, & Barch, 2005), but are not necessarily so. Reward incentives have been shown to selectively increase AY interference in this task (along with improved performance in other conditions), reflecting enhanced cue maintenance and proactive control (Chiew & Braver, 2013; Locke & Braver, 2008).

In contrast to reward, diverging accounts have been proposed to account for the effects of positive affect on cognitive-control dynamics. Ashby and colleagues (Ashby, Isen, & Turken, 1999) proposed that the effects of positive emotion on cognition are similar to those of reward, increasing DA release (via the substantia nigra and ventral tegmental area) to the anterior cingulate cortex (ACC) and striatum. However, Ashby and colleagues proposed that this DA input would facilitate goal switching and updating as opposed to maintenance, accounting for behavioral observations that positive affect may facilitate cognitive broadening/flexibility and creative problem solving (Dreisbach, 2006; Fredrickson & Branigan, 2005; Isen et al., 1987). Departing from this literature are observations from the social psychology literature that coactivation of a neutral goal concept and positive affect can lead to unconscious goal pursuit (Aarts, Custers, & Veltkamp, 2008; Custers & Aarts, 2005): These observations have led to an alternative hypothesis that positive emotion may support goal-related proactive control, even when they are not directly relevant to the goal (Aarts et al., 2008). Intriguingly, when positive-affect effects on cognition have been studied using the AX-CPT paradigm, positive affect has led to decreased proactive control and increased reactive control relative to neutral (Dreisbach, 2006), but also to decreased proactive control without significant changes in reactive control (Frober & Dreisbach, 2012; van Wouwe et al., 2011). Thus, mixed evidence has been observed with regard to positive affect’s influences on cognitive control, but overall the previous literature suggests that its effects have been distinct from those observed with reward incentives.

Emerging from these literatures are two key observations using the AX-CPT, suggesting that effects of positive affect and reward on cognition may be distinct: Reward incentives have been shown to increase AY interference in this task, reflecting enhanced cue maintenance and proactive control (Chiew & Braver, 2013; Locke & Braver, 2008); in contrast, positive-affect manipulations have reduced AY interference and, in some cases, increased BX interference, suggesting reduced proactive control, and possibly enhanced reactive control (Dreisbach, 2006). To our knowledge, these effects have not previously been compared within a single study (but see Frober & Dreisbach, 2014, in this issue). Both Chiew and Braver (2013) and Locke and Braver (2008) utilized a within-subjects design permitting examination of both block reward manipulations (i.e., a baseline vs. reward block) and trial reward manipulations (incentive and nonincentive trials randomly intermixed within the reward block), allowing relative measures of both block (tonic) and trial (phasic) effects of incentive to be indexed. In contrast, Dreisbach examined positive and neutral affect in separate groups with emotional stimuli presented on each trial; with this design, it is unclear whether observed effects were tonic or phasic in nature. The present study aims to compare these manipulations within a single group of participants and use both tonic and phasic manipulations of emotion/reward to clarify the temporal dynamics of their effects.

We used pupillometry in the present study to provide additional information and constraints on the cognitive control dynamics observed during positive affect and reward motivation conditions. The pupil demonstrates tiny, cognitively related fluctuations in diameter independent of visual luminance, which have been well-established as indexing changes in cognitive demand and effort (Beatty, 1982a, b; Granholm, Asarnow, Sarkin, & Dykes, 1996). Previous work suggested that pupillometry may also index changes in cognitive control dynamics as a result of incentive (Chiew & Braver, 2013). In particular, increased preparatory pupil activity during cue maintenance was observed under incentive relative to nonincentive conditions, prior to behavioral response. Pupil diameter is also responsive to emotional arousal associated with sympathetic nervous system activity when viewing emotionally evocative stimuli independent of a cognitive task (Bradley, Miccoli, Escrig, & Lang, 2008). Although it is not yet clear how interactions between emotion/reward and cognitive influences may impact pupil diameter, the apparent sensitivity to both factors suggests that pupillometry may be ideal for investigating the dynamics of these interactions.

Pupil activity as an index of cognitive processing can be broken down into phasic and tonic components. Phasic pupil activity refers to task-evoked pupillary responses, in which pupil dilation is time-locked and measured in response to an eliciting event and in which dilation is measured in terms of the percentage of change relative to a prestimulus baseline. Tonic pupil activity, which may reflect more sustained processes, has been measured using different methods, including pupil diameter at a preexperimental baseline (Heitz, Schrock, Payne, & Engle, 2008) or using an average pupil measure at intertrial interval (ITI) periods (as was done in Chiew & Braver, 2013). The latter method was used in the present study to compare tonic pupil activity as a function of task context (task block).

Some prior accounts have suggested that tonic and phasic pupil dilation may reflect distinct underlying mechanisms, with high tonic dilation primarily reflective of a state of high arousal, or more specifically, high tonic locus coeruleus–norepinephrine (LC-NE) activity (Granholm & Steinhauer, 2004). In one influential account, high tonic pupil dilation as an index of high tonic LC-NE would actually reflect a more exploratory cognitive mode, marked by reduced cognitive control (Gilzenrat, Nieuwenhuis, Jepma, & Cohen, 2010). However, in Chiew and Braver (2013), reward motivation was associated with both increased phasic and tonic dilation, suggesting that both could serve as markers of proactive control. The present study provided an opportunity to further investigate this issue, by comparing positive affect and reward motivation in terms of both phasic and tonic components of pupil dilation as well as their relationship to behavioral markers of proactive control.

The key aim of the present study was to test whether positive emotion and reward motivation have distinct or similar influences on cognitive control, by utilizing a tightly matched, within-subjects design in which participants performed the same task, the AX-CPT, under both emotion and motivation conditions, in separate sessions with counterbalanced order. Likewise, following Chiew and Braver (2013), within each session both trial-by-trial and block-based (contextual) manipulations of reward motivation (or positive emotion) were employed, enabling examination of both transient and sustained effects on cognitive control. Using task performance and pupillometric measures, we tested the following hypotheses based on prior experimental findings: (1) that reward incentives would be associated with enhanced proactive control (as reflected in greater AY interference and enhancement in performance in all other trial types, as predicted by findings from Locke & Braver, 2008, and Chiew & Braver, 2013) along with increased preparatory and tonic pupil dilation, and (2) that positive emotion would be associated with (a) enhanced reactive control (as reflected in reduced AY interference, along with either no change or improvement in other trial types, in line with findings by Dreisbach and Van Wouwe); (b) enhanced proactive control (as reflected in decreased AY trial performance/enhancement in performance in all other trial types, which may be consistent with Aarts’s and Custers’s findings that unconscious positive affect was associated with enhanced goal pursuit); or (c) no significant change in maintenance-related performance or pupil activity (somewhat consistent with Van Wouwe’s findings, which reached the trend level but not statistical significance). A final key feature of the study was that we employed a large sample size in order to provide adequate statistical power to detect even subtle effects, or differences between conditions.

## Method

### Participants

A group of 112 healthy young adult participants took part (61 female, 51 male; mean age 21.0 years ± 0.27). Participants were recruited from volunteer pools maintained by the Department of Psychology at Washington University in St. Louis and from the St. Louis community using posted advertisements. All participants were right-handed, had corrected-to-normal vision, and were free from psychiatric or neurological disorders. Informed consent was observed from all participants prior to participation, in accordance with the human participant guidelines established by Washington University. Participants performed the experiment for a $10/h payment, plus an additional monetary bonus in the Reward session due to reward incentives. Although participants were not informed of this until the end of the Reward session, the bonus was a fixed amount ($5). Of the 112 participants collected, 100 had usable data (i.e., attended both experimental sessions) to be included in analyses (51 female, 49 male; mean age 21.1 years ± 2.9). Within this *N* = 100, certain portions of the data were missing or not usable for every analysis, so not every participant could be used in every analysis (*N* is noted for each analysis). In particular, 20 participants had one or more runs of pupil data that were unusable due to poor data quality, but intact task performance data, leaving *N* = 80 with the full pupillometric data (42 female, 38 male; mean age 21.2 years ± 2.9). Slightly greater numbers had complete emotion-session pupil data (*N* = 92; 48 female, 44 male; mean age 21.0 years ± 2.8) and complete reward-session pupil data (*N* = 89; 44 female, 45 male; mean age 21.2 years ± 3.0).

### General study structure

**Fig. 1:**
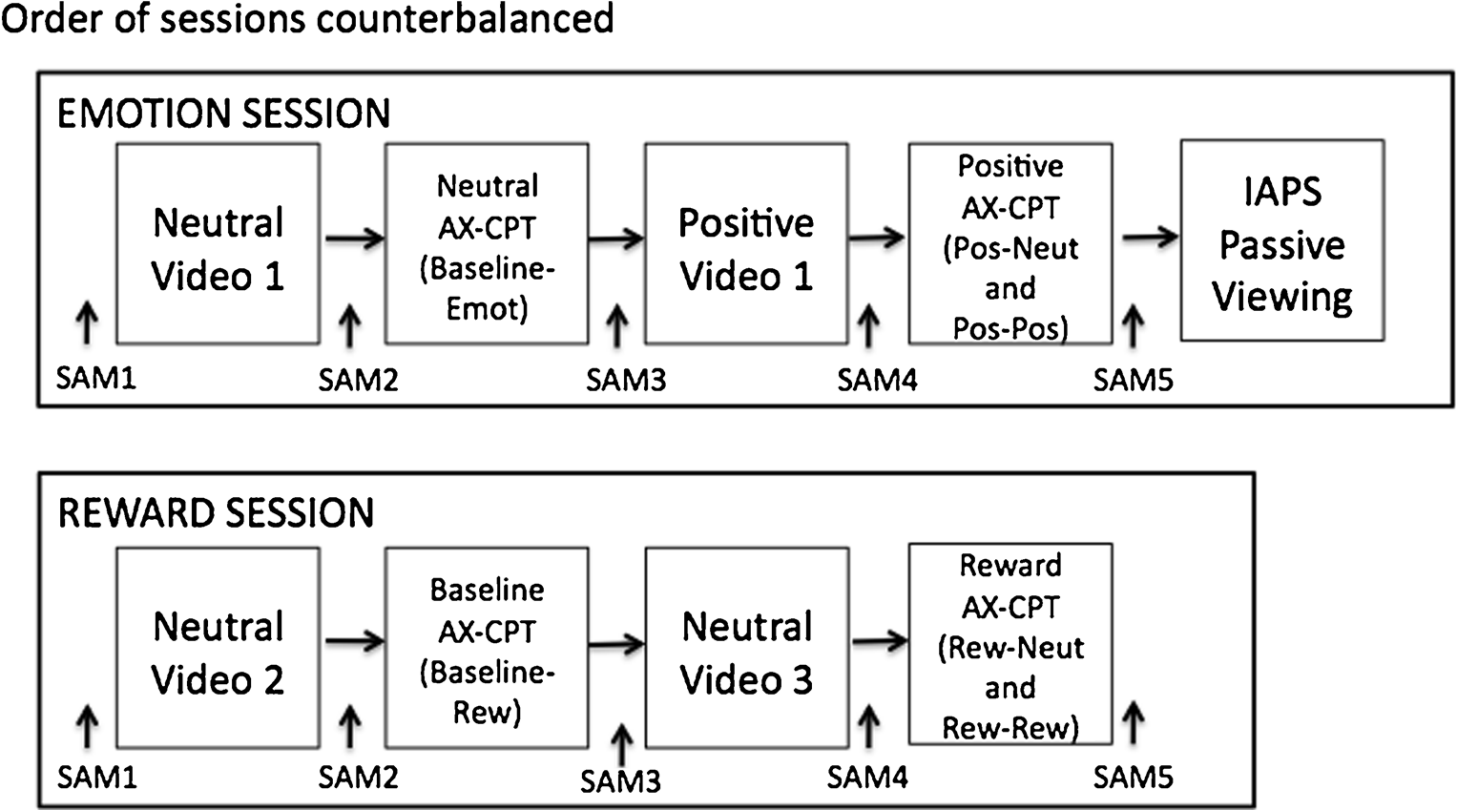
General schematic of the study structure. Participants came for two experimental sessions: the emotion session (AX-CPT under neutral and positive emotion conditions) and the reward session (AX-CPT under baseline and reward conditions). A Self-Assessment Manikin (SAM) was administered at intervals throughout the sessions, and passive viewing of IAPS images was completed following the emotion AX-CPT task runs

A general schematic of the study structure is shown in Fig. 1. Participants were seen in two separate sessions (reward and emotion, the order of which was counterbalanced), in which they completed the AX-CPT paradigm while pupil diameter data were collected using an infrared eyetracker. The two sessions were conducted on separate visits, a maximum of one week apart, and participants received $10/h pay for their participation (plus a $5 bonus at the end of the reward session, as we describe below). At the end of the two sessions, participants were fully debriefed.

In the reward session, participants performed two 200-trial blocks of the AX-CPT in a static order: one block under baseline conditions (Baseline–Rew; i.e., no reward incentives were provided), followed by one block under reward conditions (reward; i.e., reward incentives were provided). In the reward block, incentive and nonincentive trials (50% each; referred to as Rew–Rew and Rew–Neut, respectively) were randomly intermixed. Participants viewed a video clip prior to each task block; these video clips were used to match the procedure in the emotion session (see below), but had neutral valence (video stimuli from Gray, 2001, were used). During the task block, participants viewed an image from the International Affective Picture System (IAPS; Lang, Bradley, & Cuthbert, 1999) stimulus set as a precue to each trial. Two neutrally valenced IAPS images were used (chosen from the stimulus set of Dreisbach, 2006,1 with the image selection randomized across participants). Participants were told that these images were meaningless in the baseline block, whereas in the reward block one image served as an incentive cue, and one image served as a nonincentive cue, signifying the presence or absence of incentive on each trial.

In the emotion session, participants again performed two 200-trial blocks of the AX-CPT in a static order: an emotion baseline block with induction of a neutral emotion (Baseline–Emot), followed by a block under positive-emotion induction (positive). Each block was preceded by a brief video clip that was intended to induce the appropriate emotional mood [again with video stimuli taken from Gray, 2001; following the procedure used in van Wouwe et al., 2011, for the Baseline–Emot condition we used a neutral video clip, whereas for the positive condition we used a positive (or humorous) video clip]. Then, during the task block, participants viewed valenced IAPS images as a precue to each trial. This precue manipulation followed the trial-by-trial emotional stimuli used by Dreisbach (2006), in which positive and neutral emotion inductions led to differing patterns of cognitive control. In the Baseline–Emot block, all precue images were neutrally valenced, whereas in the positive block, positive and neutral precue images (50% each) were randomly intermixed (these are referred to as Pos–Pos and Pos–Neut trials, respectively).

Thus, the reward and emotions had parallel design structures, in that each session comprised a baseline block followed by an emotion/reward block, with each block being preceded by a video clip. Across the two sessions, three neutrally valenced video clips and one positively valenced video clip were used for each participant. The order of the clips was counterbalanced across participants, and no clips had repeated viewings (i.e., three different neutral clips were used for each participant). All clips were approximately 10 min in length, and no significant differences in performance were observed as a result of the counterbalancing of video clip presentations. Likewise, all AX-CPT task trials in both sessions were preceded by IAPS precues. In the baseline blocks and the reward session, these were always neutral IAPS images, whereas in the positive block, intermixed neutral and positive IAPS stimuli were used. The neutral images were repeated across the baseline and reward/emotion task blocks; positive images were novel upon first presentation, but then were repeated through the positive block. Although the emotion session was patterned off of the experimental manipulations used by van Wouwe et al. (2011) and Dreisbach (2006), it differed from both of these prior studies, since they did not manipulate emotion valence in a within-subjects manner.

As a validity check of the emotion manipulation, participants’ emotional states were assessed before and after each video clip and task block (i.e., five times in total during each experiment session) using Bradley and Lang’s (1994) Self-Assessment Manikins (SAM; a brief, nonverbal measure of the pleasure, arousal, and dominance associated with a person’s affective reaction). In each assessment, participants self-reported the valence and arousal of their present mood on a 5-point Likert scale. As a second validity check of the emotion evoked by the IAPS images, on a transient basis in the positive block, participants completed a passive viewing task of positive and neutral IAPS images (both those viewed in the AX-CPT and novel images, matched on normed valence and arousal ratings) after the AX-CPT task blocks in the emotion session. This was done to index pupil activity related to emotional arousal, independent of cognitive demands (in a manner similar to the one used by Bradley et al., 2008). A brief recognition memory test was given after the passive-viewing block. This test was implemented to ensure that participants attended to the images, but it was not scored. More specific information about SAM assessment administration and the passive-viewing run is included in the Method section of the supplementary materials.

### AX-CPT paradigm

The AX-CPT consists of a series of continuous trials in which single letters are presented in cue–probe sequences. One specific cue–probe trial sequence requires a target response (i.e., “A” followed by “X”; AX trial), with all other combinations of letters requiring a nontarget response. The AX target trial type occurs with 70% frequency and is randomly intermixed with three types of nontarget trials, each occurring with 10% frequency: AY (target cue, nontarget probe), BX (nontarget cue, target probe), and BY (nontarget cue, nontarget probe). Besides “A” and “X,” the stimuli that were used as the B and Y (nontarget) stimuli were the letters “B,” “D,” “E,” “F,” “G,” “M,” “P,” “S,” “U,” “Y,” and “Z.”

**Fig. 2:**
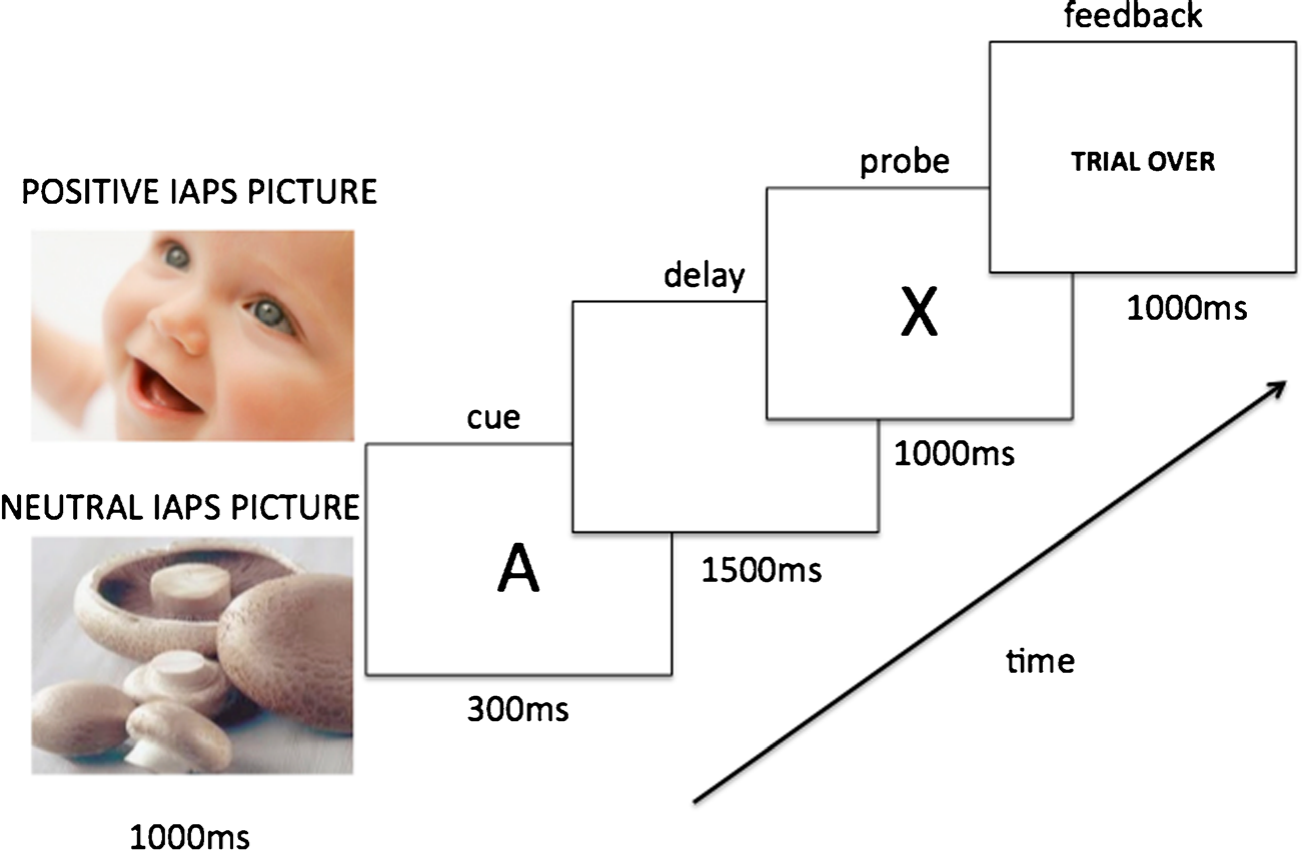
AX-CPT trial structure. In the neutral block, only neutral IAPS images were presented. In the positive block, neutral and positive IAPS images were intermixed and presented randomly on each trial. In baseline and reward trials, only two neutral IAPS images were presented per participant (chosen via random counterbalance): Participants were told in the baseline block that these images had no meaning, and in the reward block they were explicitly informed which one signified incentive and which did not

The trial structure is shown in Fig. 2. Each trial began with a 1,000-ms precue: an image from the IAPS stimulus set. Following the precue, the contextual cue (e.g., “A”) appeared for 300 ms, presented centrally in white on a black screen (Arial font, size 42). The contextual cue was followed by a 1,500-ms fixation cross, and then a probe letter appeared in the same font (e.g., the target probe “X”) for 1,000 ms, during which time the participant was required to respond to the cue–probe combination (indicating whether it was a target or nontarget trial). Following probe presentation, a feedback screen appeared for 1,000 ms. In the emotion session (for both neutral and positive blocks), the feedback message read “Trial Over” if the participant had answered correctly, and “Error” if the participant had answered incorrectly. This pattern of feedback was also provided in the Baseline–Rew block. In the reward block, the feedback pattern varied with trial incentive status. In Rew–Neut trials, feedback followed the same pattern as in the Baseline–Rew block. In Rew–Rew trials, the feedback message read “You Won a Bonus!” if the participant had replied accurately and under the reaction time (RT) cutoff (i.e., meeting the reward criteria; the cutoff was calculated for each individual participant as the fastest 30th percentile of correct baseline-block RTs), “Trial Over” if the participant had replied accurately but slower than the RT cutoff, and “Error” if the participant had made an error. Trials were separated by an ITI of 4,000 ms. This reward schedule mirrors that used in a previous study from our laboratory (Chiew & Braver, 2013).

### Experimental apparatus

The experimental paradigm was presented using E-Prime (Psychology Software Tools, Inc., Pittsburgh, PA) on a Dell PC computer. Participants were seated in a chair, with a headrest supporting the back of the head to minimize motion, and they viewed the paradigm on a computer monitor. Accuracy and RTs were collected using an E-Prime Button Box connected to the stimuli computer.

### Pupillometry data collection

Pupil data were collected as participants completed the task using an EyeLink 1000 infrared eyetracker (SR Research Ltd., Mississauga, ON) running Eyelink software (version 4.48), sampling at 1000 Hz and a spatial resolution of <.01º RMS. Calibration and validation of gaze direction were conducted before each experimental run. Pupillometry data were preprocessed using in-house software written in Java (Oracle Corp., Redwood Shores, CA). Blinks were corrected for by using linear interpolation. Only correct-response trials were included in the pupillometric analysis (there were too few errors to analyze separately). For examinations of transient (trial-related) effects, we examined each trial’s pupil activity, normalized as a percentage of change from a baseline period (the 100 ms of ITI prior to each trial onset), whereas for examinations of sustained (block-related) effects, we examined pupil activity in EyeLink’s scaled pupil diameter values rather than absolute sizes—scaled values generally range between 3,000–7,000 (corresponding to approximately 3–7 mm, following Marshall, 2007).

### Data analysis

#### Task performance

Behavioral performance data was analyzed with separate repeated measures analyses of variance (ANOVAs) conducted on error rates and median correct RTs as dependent variables. We conducted parallel analyses to compare block and trial-related effects of emotion and motivational incentive on task performance in the emotion session and reward session, respectively.

We also calculated and conducted analyses on a behavioral variable called the *proactive index* (Braver, Paxton, Locke, & Barch, 2009). The proactive index is a variable, computed from the RTs and error rates in AY and BX trials, that measures the relative tendency toward proactive control: Slower RTs/higher error rates in AY trials are considered to be indicative of proactive control, whereas slower RTs/higher error rates in BX trials are considered to be indicative of reactive control. The proactive index is a standardized score based on the relative performance on AY versus BX trials, assuming that these trial types are complementary,^2^ while normalizing across total error rates on both trials (in order to differentiate selective from general performance patterns). For RTs, the proactive index was calculated as (AY – BX)/(AY + BX). For errors, the same equation was used, but correction had to be applied when error rates were equal to zero, as follows: (error + 0.5)/(frequency of trials + 1). The proactive index calculation yields a score between –1 and +1: The closer a score is to +1, the more proactive is task performance considered to be.

To examine the block-related incentive effect on performance (in the reward session), we conducted a 2 × 2 × 2 ANOVA on Baseline–Rew and Rew–Neut trials with Block (baseline, reward), Contextual Cue (A, B), and Probe (X, Y) as within-subjects factors. By including only nonincentive trials in this analysis, one can examine the block-based effect without the contribution of trial-by-trial incentive effects. To examine the trial-based incentive effect on performance (in the reward session), we conducted a 2 × 2 × 2 ANOVA on Rew–Neut and Rew–Rew trials, with Trial Type (incentive, nonincentive), Contextual Cue (A, B), and Probe (X, Y) as within-subjects factors. We also compared proactive index measures as a function of incentive at both the block level (Baseline–Rew vs. Rew–Neut) and the trial level (Rew–Neut vs. Rew–Rew), using both RTs and error rates, using paired-samples *t* tests.

Analysis of the task performance data for the emotion session followed a similar structure. To examine the block-related mood effect on performance (in the emotion session), we conducted a 2 × 2 × 2 ANOVA on Baseline–Emot and Pos–Neut trials (i.e., preceded by a neutrally valenced IAPS image) with Block (neutral, positive), Contextual Cue (A, B), and Probe (X, Y) as within-subjects factors. To examine trial-based emotion effects on performance (in the emotion session), we conducted a 2 × 2 × 2 ANOVA on Pos–Neut and Pos–Pos trials, with Valence (neutral, positive), Contextual Cue (A, B), and Probe (X, Y) as within-subjects factors. As in the analyses with the reward-session data, we compared proactive index measures as a function of positive emotion at both the block level (Baseline–Emot vs. Pos–Neut) and the trial level (Pos–Neut vs. Pos–Pos), using both RTs and error rates, using paired-samples *t* tests.

#### Pupillometric measures

To examine the effect of the experimental manipulations on the pupillometric data, pupil activity was averaged within specific time windows during the trial and subjected to analysis. For analyses of the sustained emotional and incentive effects, pupil activity was examined during a 200-ms ITI period just prior to each trial’s onset in order to examine tonic, rather than task-performance-related, pupil activity as a function of block (comparing between Baseline–Emot and positive blocks in the emotion session and between Baseline–Rew and reward blocks in the reward session). For analyses of transient emotion and incentive effects, magnitudes were calculated for a 250-ms period of interest within the trial, during cue maintenance just prior to probe onset (referred to as *pre-probe-onset*, time points 2,550–2,800 ms). These magnitudes were used to contrast Rew–Neut and Rew–Rew trials, as well as Pos–Neut and Pos–Pos trials.

The average magnitudes of pupil dilation from these time periods of interest were examined using repeated measures ANOVAs analogous to those described previously for the behavioral performance data. However, because the analyses of transient effects examined a period prior to probe onset, the ANOVA excluded the Probe factor, because prior to probe onset, trial type could not be classified (thus, Incentive/Emotion Status and Contextual Cue were the only two factors in these analyses). Similarly, because analyses of sustained effects involved the time window prior to trial onset, only Block (Baseline–Rew vs. reward or Baseline–Emot vs. positive) was included as a factor.

## Results

### Validity of the positive emotion manipulation

Self-reported mood over the course of the experimental sessions, as indexed by SAM assessments, was consistent with the idea that the positive mood induction utilized prior to the positive block was effective. The data from the passive-viewing run of the IAPS images indicated that pupil diameter was sensitive to information presented in the images but did not significantly differentiate between different positive and neutral images, as was predicted by Bradley et al. (2008). These results are discussed further in the supplementary materials.

### Behavioral performance during AX-CPT

Critical results from analyses of the reward session (contrasting incentive and nonincentive) and the emotion session (contrasting neutral and positive emotion), along with a direct comparison of the two sessions, are described here; a more comprehensive set of analyses is reported among the Results in the supplementary materials.

#### Reward AX-CPT

The behavioral performance results from the reward session were very similar to those observed in Chiew and Braver (2013). The incentive manipulation was successful in globally improving performance: The average reward rate was 69.9% (range: 0%–94%), versus 78.5% in Chiew and Braver (2013), and versus an expected rate of 30% if performance had remained at baseline levels.

**Fig. 3:**
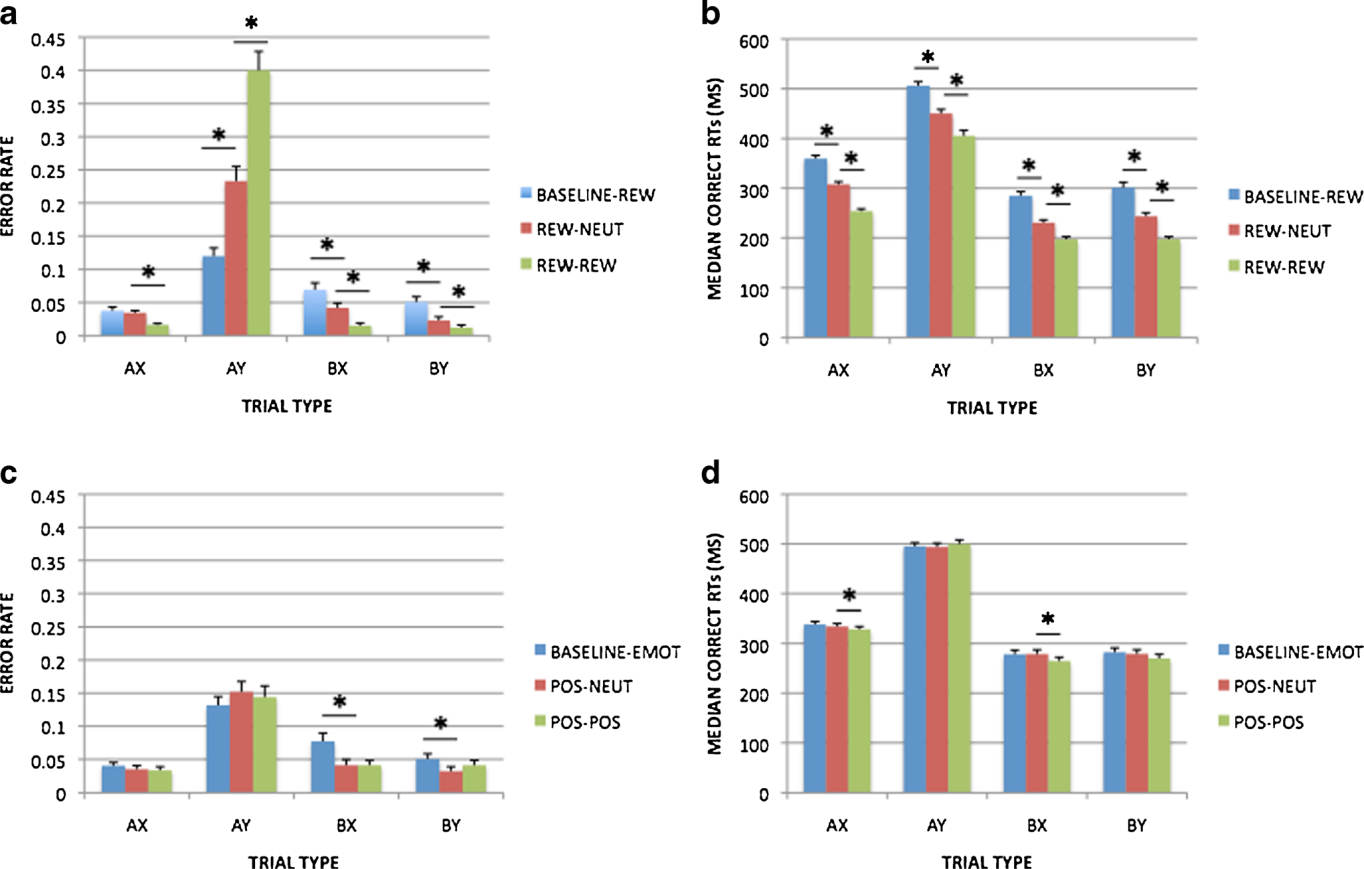
Task performance measures in the reward and emotion sessions. The top panels show trial-related incentive effects (Rew–Neut vs. Rew–Rew trials) and block-related incentive effects (Baseline–Rew vs. Rew–Neut trials) in (a) error rates and (b) RTs; the bottom panels show trial-related emotion effects (Pos–Neut vs. Pos–Pos trials) and block-related emotion effects (Baseline–Emot vs. Pos–Neut trials) in (c) error rates and (d) RTs. Significant contrasts (*p* < .05) are marked with asterisks

Block-based analyses of incentive (contrasting Baseline–Rew and Rew–Neut trials) replicated the findings in Chiew and Braver (2013) for both error and RT measures. The analysis of error rates (shown in Fig. 3a) revealed a critical Block × Cue × Probe interaction [*F*(1, 99) = 31.780, *p* < .001], due to increased AY error rates but decreased error rates in all other trial types in the reward block, relative to the baseline block. Post-hoc simple contrasts within each trial type suggested that this effect was significant for all trial types except AX (AX, *p* = .501; AY, *p* < .001; BX, *p* = .003; BY, *p* = .001). Analyses of RTs (shown in Fig. 3b) revealed a main effect of block, with speeding in the reward block relative to baseline [*F*(1, 99) = 123.171, *p* < .001], but no additional interactions of incentive with trial type (Block × Cue, *p* = .743, Block × Probe *p* = .544; Block × Cue × Probe, *p* = .993). Post-hoc simple contrasts that the speeding effect was significant in each trial type (all *p*s < .001).

Trial-based analyses of incentive (contrasting Rew–Neut and Rew–Rew trials) yielded results similar to the block-based effects and also replicated the results in Chiew and Braver (2013). In error rates (Fig. 3a), a critical Incentive × Cue × Probe interaction [*F*(1, 99) = 42.047, *p* < .001] was again observed, due to elevated AY errors and decreased errors in other trial types; post-hoc contrasts suggested that this effect was significant for all trial types (AX, *p* < .001; AY, *p* < .001; BX, *p* = .001; BY, *p* = .040). Similarly, RTs (Fig. 3b) showed the same general pattern of speeding under incentive [*F*(1, 99) = 93.942, *p* < .001] with no trial type interactions (Trial × Cue, *p* = .188; Trial × Probe, *p* = .738; Trial × Cue × Probe, *p* = .179). Post-hoc contrasts indicated that the speeding effect was significant in each trial type (all *p*s < .001).

#### Emotion AX-CPT

Block and trial-based analyses of positive emotion (contrasting Pos–Neut and Pos–Pos trials) were conducted in a similar fashion to those in the reward session. In error rates (Fig. 3c), the block-based analyses of positive emotion effects indicated a significant Block × Cue interaction [*F*(1, 99) = 10.755, *p* = .001] and a Block × Probe interaction [*F*(1, 99) = 4.927, *p* = .029]. These effects were due to elevated AY errors and decreased errors in all other conditions in the positive block relative to neutral. This pattern was similar to, but much more subtle than that observed under reward, and thus did not support a significant three-way Block × Cue × Probe interaction (*p* = .696). Indeed, simple contrasts showed that the positive-emotion effect was only significant for B-cue trials (AX, *p* = .260; AY, *p* = .156; BX, *p* = .007; BY, *p* = .030). For RTs (Fig. 3d), we observed no significant emotion block effects (for all block-related effects and interactions, *F*s < 1).

Trial-based analyses of positive emotion (contrasting Pos–Neut and Pos–Pos trials) did not reveal any significant effects of emotion on error rates (Fig. 3c; for all trial-related effects and interactions, *F*s < 1). In RTs (Fig. 3d), a trend-level main effect of emotion [*F*(1, 99) = 3.771, *p* = .055] and a significant Emotion × Cue interaction [*F*(1, 99) = 5.175, *p* = .025] were observed. These results were due to elevated RTs in AY trials and decreased RTs in all other trial types in Pos–Pos versus Pos–Neut trials, which follows the pattern associated with a shift toward greater proactive control, albeit a small one (<10 ms for all four trial types). Post-hoc contrasts confirmed the subtlety of the effect, since only X-probe trial types were significant (AX, *p* = .018; AY, *p* = .380; BX, *p* = .013; BY, *p* = .114).

### Proactive-control focused analyses and comparisons

**Fig. 4:**
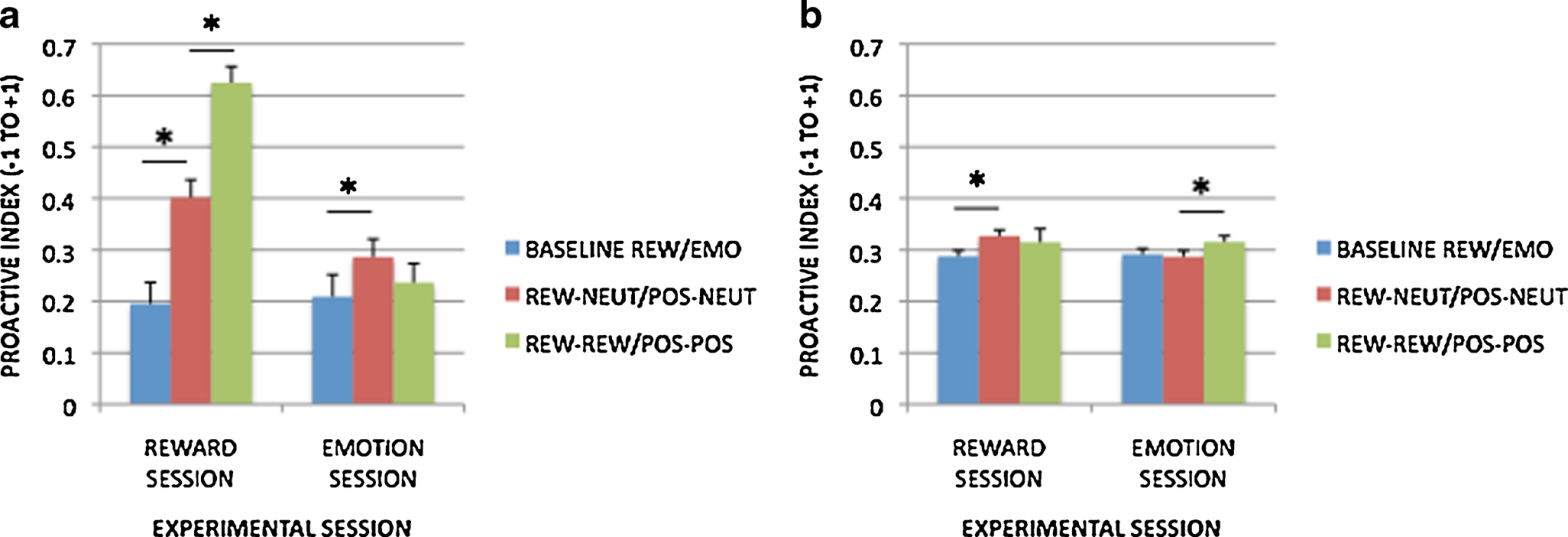
Proactive index measures in each condition for the reward session (Baseline–Rew, Rew–Neut, Rew–Rew) and the emotion session (Baseline–Emot, Pos–Neut, Pos–Pos): (a) calculated with error rates; (b) calculated with RTs. Significant contrasts (*p* < .05) are marked with asterisks

A potentially more powerful and sensitive means of examining the effects of reward motivation and positive affect on cognitive control is through the proactive control index (see the Method section and Braver et al., 2009, for details), which was applied to both the error and RT measures. Graphs of the proactive index measures are shown in Fig. 4 (4a for error rates and 4b for RTs). For reward motivation, block-based incentive effects were associated with higher proactive indices for both error rates [*t*(99) = 3.413, *p* = .001] and RTs [*t*(99) = 4.673, *p* < .001]. The latter effect supports the utility of the proactive control index, since a similar pattern was not observed in the raw RT data (i.e., the lack of interaction with trial type). Trial-based incentive effects also revealed higher proactive indices for error rates [Rew–Rew > Rew–Neut; *t*(99) = 5.339, *p* < .001], but not for RTs [*t*(99) = –0.490, *p* = .625].

When examining the positive-emotion condition, we found no significant changes in the proactive index in terms of block-based effects [errors, *t*(99) = 0.466, *p* = .642; RTs, *t*(99) = –1.590, *p* = .115]. Likewise, when considering trial-based effects, no proactive increase was apparent for error rates (*p* = .252), although a weak effect was observed for RTs [*t*(99) = 2.528, *p* = .013].

These patterns suggest that the reward and emotion session data both yielded a weak shift toward proactive control, but that this effect was much more robust within the reward condition. To test this assertion, we directly contrasted the two conditions in a series of 2 × 2 ANOVAs. In the block-based comparison (a 2 [condition: emotion, reward] × 2 [block contrast: Baseline–Rew vs. Rew–Neut or Baseline–Emot vs. Pos–Neut]) ANOVA, the analysis revealed significant effects of block [*F*(1, 99) = 17.008, *p* < .001] and Condition × Block [*F*(1, 99) = 4.421, *p* = .038] for errors; for RTs, condition [*F*(1, 99) = 4.510, *p* = .036], Condition × Block [*F*(1, 99) = 10.248, *p* = .002], and trend-level block [*F*(1, 99) = 3.787, *p* = .055] effects were observed. In the trial-based comparison (a 2 [condition: emotion, reward] × 2 [trial contrast: Rew–Neut vs. Rew–Rew or Pos–Neut vs. Pos–Pos]), the ANOVA revealed significant effects of condition [*F*(1, 99) = 51.118, *p* < .001], trial [*F*(1, 99) = 7.335, *p* = .008], and Session × Trial [*F*(1, 99) = 25.257, *p* < .001] for errors; for RTs, no significant effects were observed [session, *F*(1, 99) = 1.407, *p* = .238; trial, *F*(1, 99) = 0.367, *p* = .546; Session × Trial, *F*(1, 99) = 2.496, *p* = .117].

Together, these patterns provide strong confirmation for the hypothesis that proactive control is increased with reward motivation; the behavioral data further suggested that this pattern was present in terms of both block-based (contextual) and trial-by-trial motivational manipulations. Moreover, a direct comparison of the conditions indicated that proactive control was significantly greater under reward than under emotion, although for the trial-based manipulations, the Reward > Emotion effect was only present in errors and not RTs. Most importantly, the emotion session effects did not provide any evidence for the hypothesis that reactive control would increase under positive emotion; instead, positive emotion appeared to be associated with a weak shift toward proactive control, primarily observed as a block-based, or contextual, effect.

### Pupillometry measures during the AX-CPT

Analyses of the pupillometry data paralleled the analysis of behavioral performance measures. Block- and trial-based effects in the reward and emotion session data were analyzed separately and will be briefly described, whereas the comparison of reward and emotion effects across sessions will be focused upon in more detail.

#### Reward AX-CPT

**Fig. 5:**
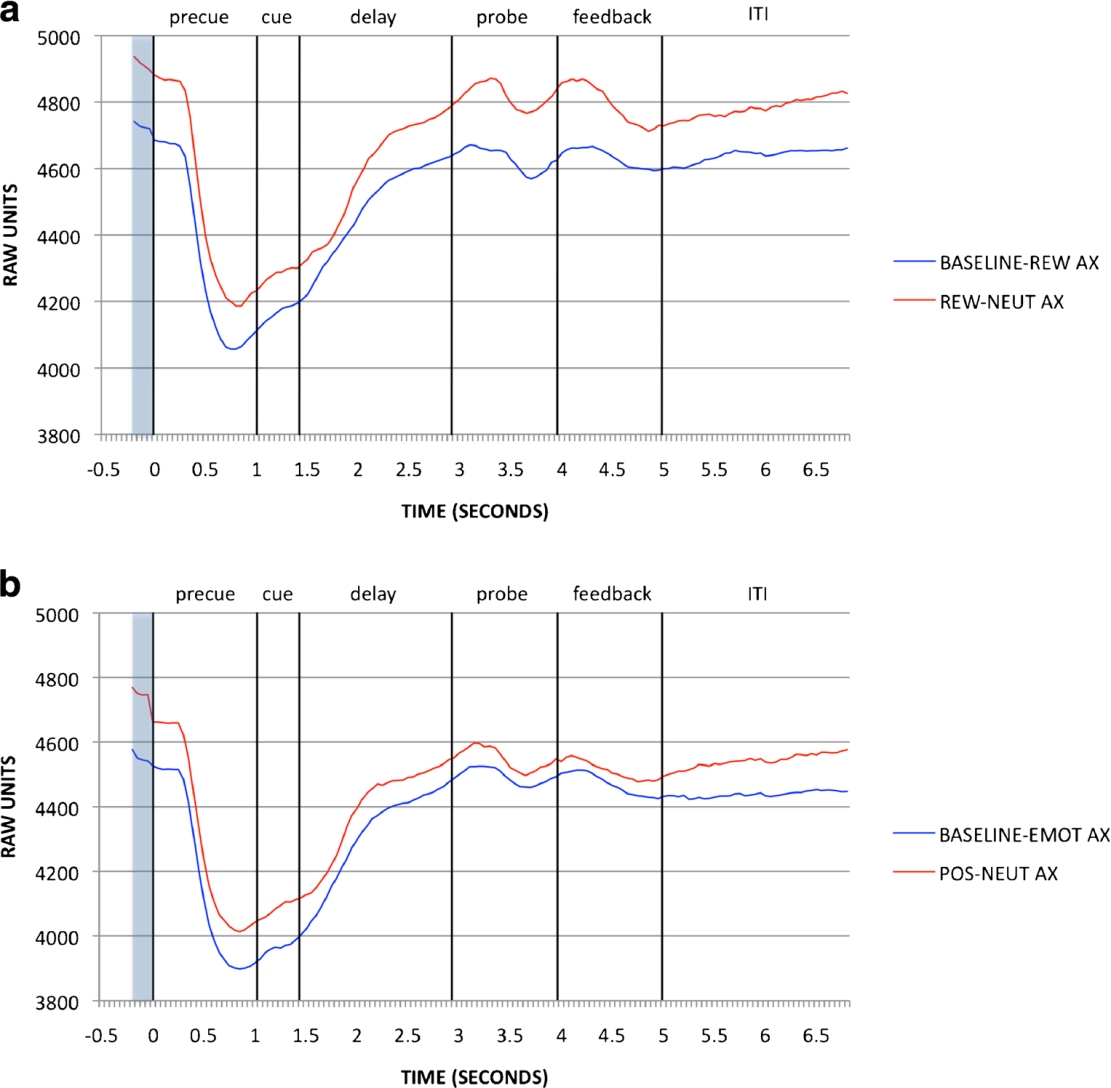
Pupil time courses for the block-based incentive contrast in (a) the reward session (Baseline–Rew vs. Rew–Neut) and (b) the emotion session (Baseline–Emot vs. Pos–Neut). The pretrial period (–200 to 0 ms), during which pupil magnitudes were analyzed for this contrast, is shaded

**Fig. 6:**
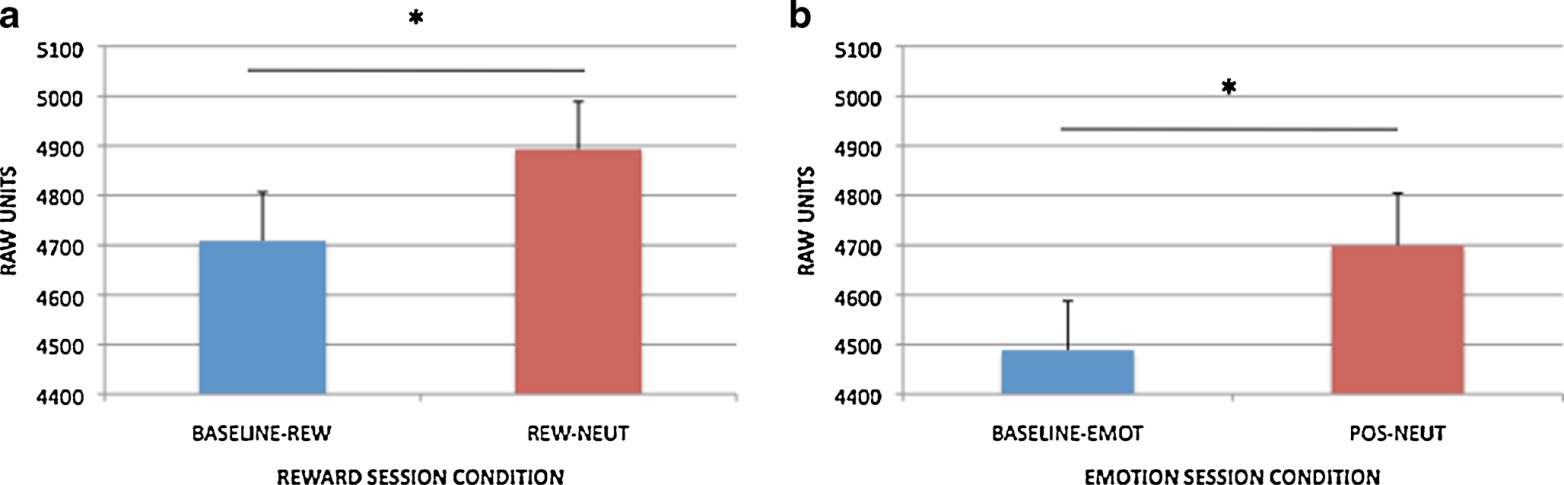
Block-based incentive (reward session data) and emotion (emotion session data) effects, as averaged pupil magnitudes in the pretrial period (–200 to 0 ms). Significant contrasts (*p* < .05) are marked with asterisks

**Fig. 7:**
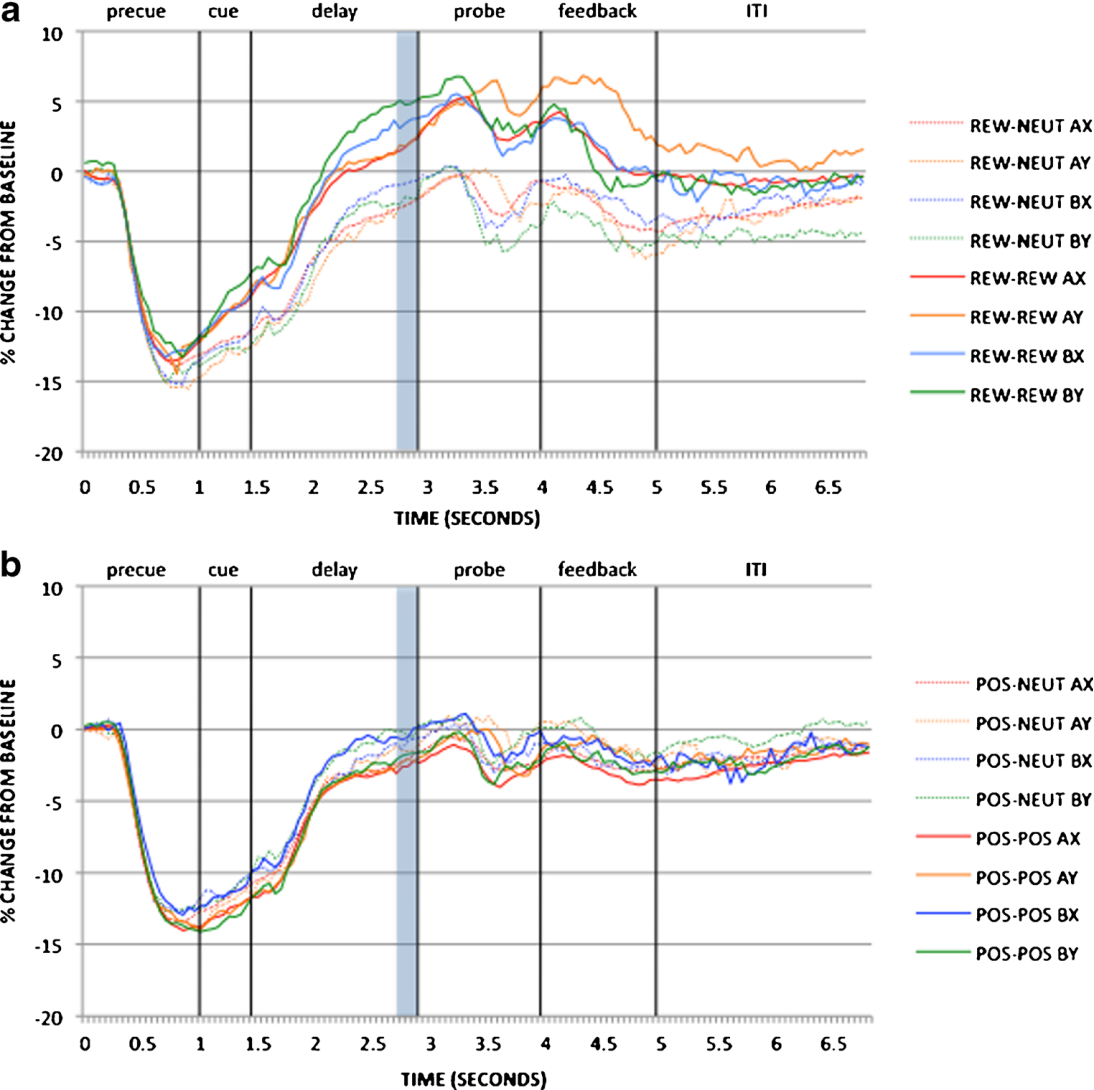
Pupil time courses for the trial-based incentive contrast in (a) the reward session (Rew–Neut vs. Rew–Rew) and (b) the emotion session (Pos–Neut vs. Pos–Pos). The cue maintenance period (2,550–2,800 ms), during which pupil magnitudes were analyzed for this contrast, is shaded

**Fig. 8:**
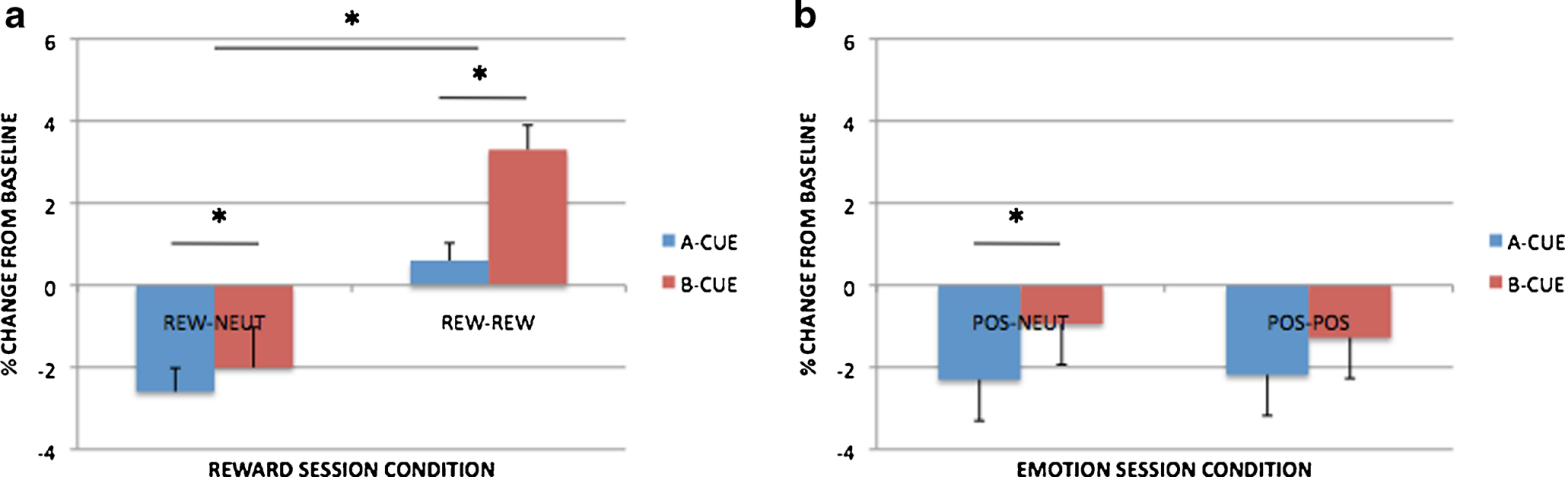
Trial-based incentive (reward session data) and emotion (emotion session data) effects as averaged pupil magnitudes in the cue maintenance period (2,550–2,800 ms). Significant contrasts (*p* < .05) are marked with asterisks

As we described in Method section, block effects of incentive on pupil activity in the reward session were examined at the pretrial period: Replicating Chiew and Braver (2013), dilation was greater in the reward than in the Baseline–Rew block [*t*(88) = 4.102, *p* < .001; full time courses for the AX trials are shown in Fig. 5a, and pretrial effects are in Fig. 6a]. Trial-based effects of incentive on pupil were examined during cue maintenance, as described in the Method section, in Rew–Neut and Rew–Rew trials. Pupil activity was also examined as a function of cue (A, B). These effects are shown in Figs. 7a and 8a. Within the maintenance period, pupil dilation was greater in Rew–Rew than in Rew–Neut trials [*F*(1, 88) = 25.159, *p* < .001] and in B- than in A-cue trials [*F*(1, 88) = 19.955, *p* < .001]; a significant Incentive × Cue interaction [*F*(1, 88) = 7.568, *p* = .007] indicated that the B > A cue effect was amplified under incentive. This pattern replicates previous results (Chiew & Braver, 2013), supporting the notion that pupil activity may index greater effort with incentive and with B, versus A, cues. Intriguingly, the B > A pattern suggests that greater preparatory effort is exerted in nontarget trials than in target trials: In nontarget trials the cue may have higher utility, since the dominant target response bias must be overcome (i.e., target responses were required on 70% of all trials, and on 87.5% of the A-cue trials, but on 0% of B-cue trials).

#### Emotion AX-CPT

Analyses of block- and trial-based emotion effects in pupil data followed the structure used for the reward session data. In the block contrast, pretrial pupil magnitude was significantly greater in the positive block than the Baseline–Emot block [*t*(91) = 3.242, *p* = .002; full time courses for AX trials are shown in Fig. 5b, with pretrial effects in Fig. 6b]. Although this effect is similar to that observed in the reward session, overt task performance in the Baseline–Emot versus positive block was much less differentiated than that between baseline and reward. Block-based emotion effects in the pupil may thus reflect increased arousal in the positive-emotion context relative to neutral emotion, rather than greater cognitive effort. Given that the positive/reward block always followed the baseline block in both sessions, it is also possible that the difference in pupil dilation could be due to order/practice effects, but supplementary time-on-task analyses provided no evidence to support this alternative interpretation (please refer to the supplementary materials). The analysis of pupil dilation during the cue maintenance period (Figs. 7b and 8b), to examine trial-based emotion effects, again yielded a B > A cue effect [*F*(1, 91) = 7.526, *p* = .007] but no significant effects of emotion condition on transient pupil activity.

#### Emotion versus reward AX-CPT

Directly building on the previous analyses of block- and trial-based effects, we compared the reward and emotion manipulations, operating at both the block and trial levels, on pupil dilation. Block-based effects were examined in pretrial pupil data as a function of session (emotion, reward) and block (first, second): The ANOVA revealed significant effects of session [*F*(1, 79) = 7.344, *p* = .008; greater pupil dilation in the reward session overall] and block [*F*(1, 79) = 31.463, *p* < .001; greater pupil dilation in the reward/positive block than in Baseline–Rew or Baseline–Emot], but no significant Session × Block interaction. This suggests that, although overall tonic pupil activity was elevated in the reward session relative to the emotion session, the block-based increases in pupil dilation in response to reward/emotion manipulations were comparable.

Trial-based effects of emotion and reward were examined in pupil dilation at cue maintenance as a function of session (emotion, reward), trial (Pos–Pos/Rew–Rew vs. Pos–Neut/Rew–Neut), and cue (A, B): This analysis yielded a critical Session × Trial × Cue interaction [*F*(1, 81) = 7.475, *p* = .008], driven by the fact that pupil dilation was much higher in Rew–Rew B-cue trials than in any other condition. Significant main effects of session [*F*(1, 79) = 8.138, *p* = .006; Reward > Emotion], trial [*F*(1, 79) = 14.540, *p* < .001; Pos–Pos/Rew–Rew > Pos–Neut/Rew–Neut], cue [*F*(1, 79) = 28.437, *p* < .001; B > A], and Session × Trial [*F*(1, 79) = 27.945, *p* < .001; trial effects greater in the reward session] were also observed.

Post-hoc analyses contrasting pupil dilation in Rew–Neut versus Pos–Neut trials (× Cue) did not lead to any significant effects, showing that transient pupil activity in this control-trial condition was similar across sessions, whereas contrasting pupil dilation in Pos–Pos versus Rew–Rew trials (× Cue) revealed a significant effect of session (*p* = .002) and a significant Session × Cue interaction (*p* = .021), verifying that significant differences as a result of session were driven specifically by performance on reward/positive trials (and most amplified for B cues).

Given the presence of both block- and trial-based incentive and emotion effects in pupil activity, possible interactions between these dynamics were also explored by analyzing transient pupil activity as a function of session and block contrast. This analysis revealed that, relative to the baseline blocks, phasic dilation was reduced (along with greater tonic dilation) in both the Rew–Neut and Pos–Neut trials (these results are reported in detail in the supplementary materials). Interestingly, this pattern of increased tonic–decreased phasic dilation was opposite to that predicted by theories linking pupil activity to LC-NE function (Gilzenrat et al., 2010), since such a pattern would be expected under conditions of reduced task engagement (i.e., increased exploration). However, in the present results, this pattern of pupil dynamics was associated with behavioral performance signatures of increased proactive control (i.e., more focused task engagement; exploitation).

### Pupil–behavior correlations

To follow up the analyses examining the effects of our experimental manipulations on performance and pupil activity, we conducted pupil–behavior correlations to clarify whether pupil activity, as a putative measure of cognitive effort, could be directly related to overt behavior, and whether this relationship changed under reward and emotion conditions. We specifically tested the hypothesis that pupil activity in the cue maintenance period (2,550–2,800 ms, just prior to probe onset) and in the pretrial period (–200 to 0 ms, just prior to trial onset) would positively correlate with proactive control, and that these correlations might strengthen with incentive. These hypotheses assume that pupil dilation is a measure of cognitive effort, but are also based on observations that pupil dilation is enhanced under incentive, even when overt performance is matched to that in nonincentive trials (Chiew & Braver, 2013).

To measure the relationship between pupil dilation and proactive control, we used the proactive index as a behavioral measure, calculated using both errors and RTs, in Rew–Neut and Rew–Rew trials in the reward session, and Pos–Neut and Pos–Pos trials in the emotion session. Furthermore, we used these indices to calculate difference scores, Rew–Difference and Pos–Difference (i.e., the extents to which proactive control increased under trial manipulations of incentive/positive emotion in Rew–Rew and Pos–Pos, relative to Rew–Neut and Pos–Neut, respectively). These measures were correlated with pupil magnitudes in the cue maintenance period (measured, as before, as the percentage change from baseline) and the pretrial period. Because pupil activity in the pretrial period was calculated in absolute values, we calculated measures of dilation change between the conditions to correlate with task performance. Furthermore, given that the pretrial pupil effects were block-related in nature, we also correlated performance and pupil activity in the block contrast. Thus, we calculated a difference score of the Rew–Neut–Baseline proactive indices to correlate with Rew–Neut–Baseline pretrial pupil activity. Because of the sensitivity of the correlation analyses to outliers, outliers in the distributions were identified using the extreme Studentized deviate (ESD) method (Grubbs, 1969) and were eliminated from the data.

**Fig. 9:**
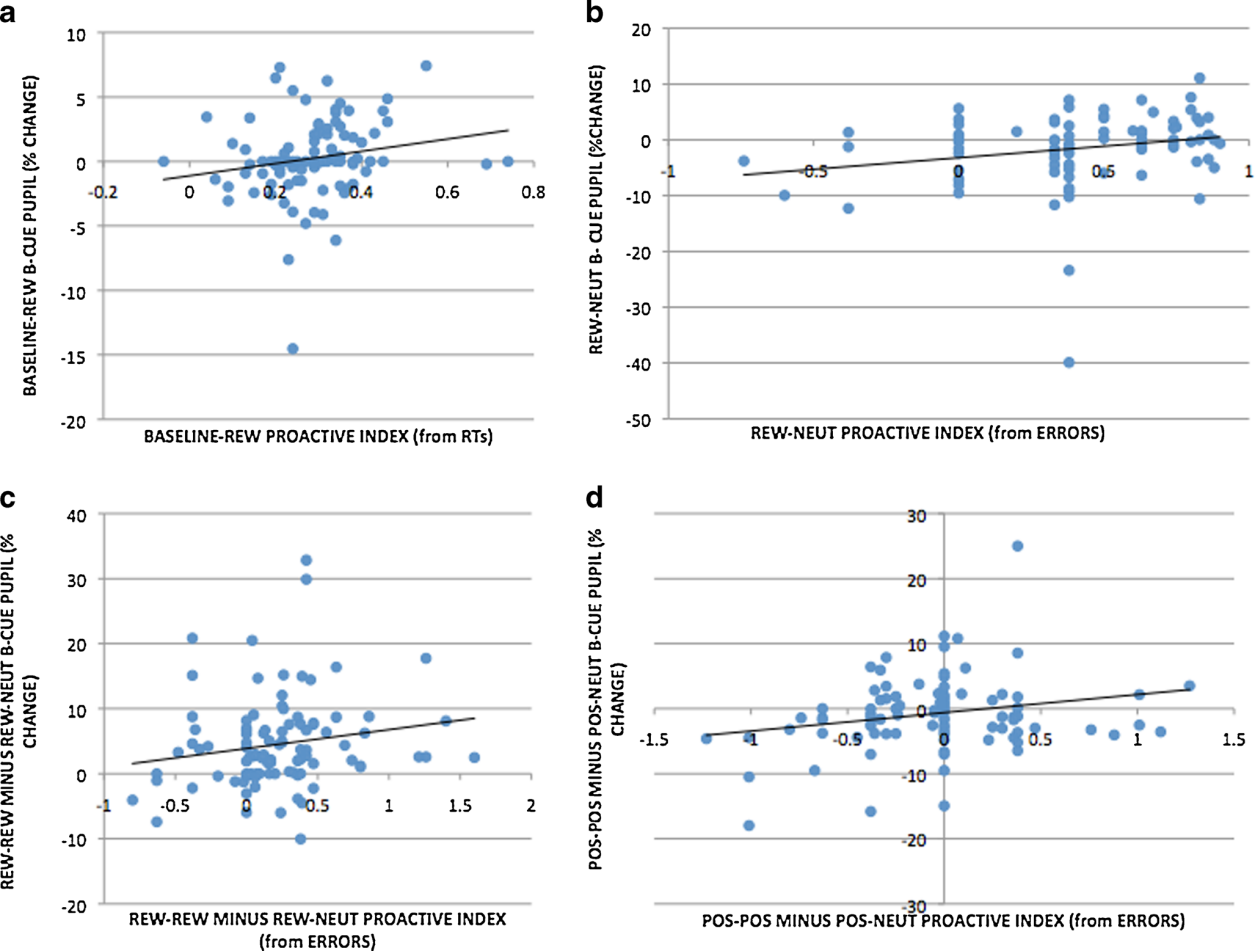
Scatterplots showing significant and trend-level pupil–behavior correlations: (a) Baseline–Rew RT proactive index versus pupil dilation for B trials (*p* = .04); (b) Rew–Neut error proactive index versus pupil dilation for B cues (*p* = .024); (c) Rew–Rew minus Rew–Neut proactive indices (from errors) plotted against pupil dilation for B cues (*p* = .085); (d) Pos–Pos minus Pos–Neut proactive indices (from errors) plotted against pupil dilation for B cues (*p* = .053)

Of the correlations examining the relationship between proactive indices and pupil dilation in the cue maintenance period, we observed significant positive correlations between the Baseline–Rew RT proactive index and Baseline–Rew B-cue pupil dilation [*r*(98) = .208, *p* = .04] and between the Rew–Neut error proactive index and Rew–Neut B-cue pupil dilation [*r*(99) = .227, *p* = .024], as well as a positive trend-level correlation between the Rew–Rew minus Rew–Neut error proactive index and Rew–Rew minus Rew–Neut B-cue pupil dilation [*r*(99) = .174, *p* = .085]. Patterns in the emotion-session data showed a similar but weaker pattern, with trend-level positive correlations being observed between the Pos–Pos minus Pos–Neut error proactive index and Pos–Pos minus Pos–Neut B-cue pupil dilation [*r*(98) = .184, *p* = .053]. Scatterplots of these relationships are shown in Fig. 9.

These findings provide support that phasic pupil dilation in the cue maintenance period may reflect proactive control, including evidence, albeit at a trend level, that incentive- and emotion-related increases in pupil dilation may predict corresponding increases in proactive control. No significant correlations were observed between proactive indices and pupil activity in the pretrial period in either the reward or emotion session. The latter pattern of null effects is consistent with the interpretation that tonic pupil dilation may serve as a marker of general arousal rather than of proactive control, per se.

## Discussion

Influences of positive emotion and reward incentives have shown diverging effects on cognitive control in previous literature, but these effects have yet to be directly compared in a study with matched experimental designs. The present study addressed this issue, using behavioral performance and pupillometry to index changes in cognitive control dynamics. In this investigation, we aimed not only to replicate previous findings that reward incentives increased proactive control (Chiew & Braver, 2013), but most importantly, to directly compare effects of positive emotion and reward incentive on cognitive control. We will discuss the findings, the implications and limitations of the present data, and directions for future research.

The results from the reward session closely replicated those of previous studies in indicating that incentive enhanced proactive control, relative to baseline (Chiew & Braver, 2013; Jimura et al., 2010; Locke & Braver, 2008; Padmala & Pessoa, 2011). Incentive effects occurred in both block- and trial-based contrasts, enhancing performance in all trial types except AY, which was characterized by sharp increases in errors with both block and trial manipulations; this pattern is consistent with increased proactive utilization of contextual cue information. Incentive-related changes in pupil activity also replicated Chiew and Braver (2013): Increases in both transient and sustained pupil dilation were observed. Importantly, increases in transient dilation emerged during the cue maintenance phase—that is, in a preparatory manner prior to behavioral response.

Our interpretation that the behavioral changes occurring in the reward condition reflected enhanced proactive control was supported by the proactive index effects, which significantly increased with both block and trial-based incentive. However, an alternative interpretation of AX-CPT interference effects is that they reflect episodic binding patterns between stimulus and response, in addition to proactive control (van Wouwe, Band, & Ridderinkhof, 2009). This interpretation seems unlikely for our data, which showed very low levels of BX interference, as compared to AY interference, particularly under incentive conditions. If AX-CPT performance is completely driven by top-down, proactive control, BX interference should be almost absent, given the predictive power of the nontarget probe. In contrast, according to the episodic binding account the stimulus–response association established between the X-probe and the target response (by high-frequency AX trials) should contribute to BX interference, even when proactive control is high. Given the low BX performance costs, we suggest that stimulus–response binding effects were a relatively minor contribution to performance, and that incentive-related modulation of performance was largely driven by changes in proactive control.

Prior evidence regarding positive emotion on cognitive control has been more mixed. We thus identified three possible, tentative hypotheses regarding positive emotion to test: that positive emotion may (1) enhance reactive control and decrease proactive control; (2) result in a null effect on performance (and potentially pupil dilation as well); and (3) enhance proactive control (both behaviorally and in terms of pupillometric cue-maintenance effects). The results suggest that the positive emotion manipulation had much weaker effects on cognition than reward, and instead of enhancing reactive control, led to a small shift toward proactive control. In the trial-based emotion contrast (Pos–Neut vs. Pos–Pos), RTs showed a pattern consistent with increased proactive control (slowing in AY but speeding in all other trial types, although the overall effect was very small, <10 ms). We also observed higher AY errors and lower errors in other trials in the block-based contrast (Baseline–Emot vs. Pos–Neut) but this effect did not reach significance when analyzed as proactive indices. Furthermore, transient pupil activity did not significantly change with trial-by-trial emotion manipulation. Finally, although tonic pupil dilation did increase in the positive block relative to neutral, these changes in pupil activity did not neatly correspond to changes in task performance.

The small shift toward proactive control we observed under positive emotion, relative to neutral, is consistent with a recent study demonstrating that positive affect may promote cognitive stability, specifically when it is noncontingent in nature (Braem et al., 2013). Braem and colleagues also demonstrated that positive affect may promote flexibility when the affect is performance-contingent in nature. In important contrast to the present study, affective stimuli were used as trial feedback, as opposed to our design, in which they were presented prior to trial performance. Braem and colleagues interpreted their observations—specifically increased flexibility in the positive, performance-contingent condition—as consistent with Carver’s (2003) suggestion that increased attentional shifting (i.e., “coasting”) may occur following postgoal positive affect, whereas noncontingent appetitive positive stimuli (i.e., images of desserts) may have engendered approach motivation and promoted task focus (Gable & Harmon-Jones, 2008). Other work has shown that pregoal, relative to postgoal, positive affect (both manipulations engendered with monetary reward), may similarly promote task focus and local (vs. global) processing (Gable & Harmon-Jones, 2011). Given that affective stimuli were presented prior to each task trial in the present study, similar “pregoal” processing may have occurred. However, the extent to which our positive stimuli (intended to be nonappetitive and moderate in arousal; see Table S1 in the supplementary materials), relative to reward anticipation, specifically engendered appetitive motivation is not clear. Taken together with previous observations, our findings illustrate that effects of positive stimuli on cognition may be highly variable as a function of performance contingency, pre-/postgoal status, and stimuli content; this remains an area for future investigation.

Consistent with Chiew and Braver (2013), the present data indicates that both tonic and phasic pupil activity show sensitivity to motivational influences. Phasic pupil dilation during cue maintenance increased with reward incentives, particularly on B-cue trials, which are the most informative with regard to upcoming control demands and response preparation. The pupil–behavior correlations provided convergent results, indicating that individual differences in behavioral indices of proactive control could be predicted by preparatory pupil dilation increases (i.e., during the cue maintenance period), again particularly on B-cue trials. Together, these results suggest that B-cue pupil dilation during preparatory periods may serve as a potential psychophysiological marker of the engagement of proactive control processes and their modulation by incentive. It should be noted that given the relatively low frequency of B-trials in the present paradigm (20%), it currently remains ambiguous whether the B > A dilation pattern is due to predictive validity or low frequency. AX-CPT variants that control for frequency and predictive validity of the A and B cues (Redick, 2014) could be combined with pupillometry in the future to clarify the nature of this effect.

Influences of the positive emotion manipulation are less clearly interpretable as operating on tonic and phasic time scales. As we noted in the Results section, and elaborate upon in the supplementary material Results, passive viewing of positive and neutral images did not significantly differentiate pupil activity and, by extrapolation, emotional arousal (as predicted by findings in Bradley et al., 2008). Thus, it is possible that our transient emotional manipulation, constructed to parallel the transient manipulation of incentive, did not induce desired transient positive affect. Thus, the null effects of trial-level emotion on AX-CPT performance and pupillometric indices should be interpreted with caution. To our knowledge, the use of a combined sustained/transient design has not been previously used to induce positive affect and the validity of the manipulation is relatively unknown; thus, further investigation will need to clarify the extent to which such a design can effectively induce positive affect on both sustained and transient timescales.

We did observe that tonic pupil activity increased under both positive emotion and reward manipulations. Although the increases in tonic pupil activity were comparable in both sessions, the block-based behavioral performance effects were much larger under the reward manipulation than the emotion manipulation. Taken collectively these patterns of pupil activity are consistent with Granholm and Steinhauer (2004), who suggest that phasic pupil activity may more directly reflect cognitive effort, whereas tonic pupil activity may reflect nonspecific influences such as arousal or mood, which may not necessarily be directly linked to cognitive performance. Taken in sum, these results clearly indicate that the pupil signal is more complex than a simple indicator of mental effort *or* arousal alone. These findings add to a growing literature indicating possible pupil sensitivity to interacting influences including cognitive effort, autonomic arousal, motor preparation, and anxiety (Bertrand, Garcia, Viera, Santos, & Bertrand, 2013; Bradley et al., 2008; van Steenbergen & Band, 2013); relative contributions of each possible influence to pupil signal may also vary strongly with psychological context. Neuroimaging methodologies permitting time-locking of pupil and brain activity will be useful in disentangling these relationships.

### Neurobiological implications of observed reward/emotion effects

The present results provide robust evidence that reward incentive affects cognitive control more than positive emotion, but the neurobiological basis for this pattern of results remains unclear. A wealth of data has implicated the DA system as critical to motivation and reward processing as well as cognitive control. Although it has been argued that DA may also underlie positive affect (Ashby et al., 1999), it remains unclear whether reward and positive affect elicit DA activity in the same way. Effects of DA release on PFC and cognition are complex, involving multiple factors, including temporal dynamics and receptor activity. For instance, phasic DA activity at D1 receptors has been associated with cognitive maintenance, whereas tonic DA activity at D2 receptors has been associated with cognitive flexibility and updating (Aboitiz, 2009; van Holstein et al., 2011). Positive emotion and reward effects may have both been due to DA activity, but in different modes. Such differences may also account for heterogeneity in observations regarding the effects of positive emotion on cognitive control. It should be noted that DA’s involvement in positive emotion has been questioned (i.e., Berridge & Robinson, 1998) and L-DOPA, a DA precursor, did not elevate mood in a recent study (Liggins, Pihl, Benkelfat, & Leyton, 2012). Thus, at present, DA involvement in positive mood remains to be confirmed and clarified.

Replicating Chiew and Braver (2013), reward incentives modulated both tonic and phasic pupil activity. Given the key role of the LC-NE system in modulating arousal and pupil dilation, NE needs to be considered in these interactions, in addition to DA. Tonic and phasic norepinephrine have been related to exploration and exploitation, indexed by inversely related tonic and phasic pupil activity (Aston-Jones & Cohen, 2005; Gilzenrat et al., 2010; Murphy, Robertson, Balsters, & O’Connell R, 2011). Our data are somewhat consistent with evidence of inverse correlation between tonic and phasic pupil activity, under both reward and emotion manipulations (examining pupil activity in Baseline–Rew vs. Rew–Neut and Baseline–Emot vs. Pos–Neut data contrasts). However, adaptive gain theory predicts that a pattern of high tonic/low phasic pupil activity should be predictive of increased task exploration and decreased task engagement. Although the high tonic/low phasic pattern was observed in Rew–Neut trials, it was associated with a behavioral shift toward enhanced proactive control (i.e., increased task engagement) relative to baseline. A similar shift in error rates was observed in Pos–Neut trials relative to baseline, although this effect was much smaller. Furthermore, Rew–Rew trials were characterized by high tonic *and* phasic pupil activity, and were associated with the highest levels of proactive control.

Given that adaptive gain theory predicts that low tonic, rather than high tonic, LC-NE activity should characterize motivated performance (i.e., enhanced task engagement), this pattern of pupil activity may reflect other possible influences (i.e., reward-related dopamine release) in addition to LC-NE system activity. Pos–Pos trials showed relatively little phasic pupil or behavior change relative to Pos–Neut. Pupil activity in the Emotion session was thus more characteristic of an inverse relation between tonic and phasic pupil activity (and by extrapolation, NE), following Gilzenrat et al.’s (2010) observations. This raises the interesting possibility that pupil dilation may be sensitive to complex interactions of multiple neurotransmitter systems (i.e., NE, DA), whose relative contributions may depend on psychological context.

### Experimental limitations

Some uncertainty exists regarding the reliability and validity of the positive emotion manipulations used in the present study. Self-report of mood valence via the SAM indicated that participants noted a mild increase in mood valence after viewing the positively valenced video, relative to before the video. However, as discussed in the supplementary materials, self-reported mood valence also tended to decrease over the course of the positive task block. Similar decreases in mood valence were observed for all other task blocks, regardless of intended emotion. Mood may have become less positive over time owing to mild fatigue and/or mind-wandering, which may be associated with negative thought content (Kane et al., 2007). Because the positive block always followed the neutral block (matching the reward session), the combination of this decline with successful positive mood induction meant that reported mood valences prior to the neutral and the positive blocks were relatively similar (*p* = .364). In contrast, in the reward session, in which both blocks were preceded by neutral videos, reported mood valence was significantly higher prior to the baseline block versus reward block (*p* = .02).

Time-on-task analyses (in the supplementary materials) revealed that errors increased over the course of each task block, also consistent with the possibility of increasing fatigue over time. These results suggest that fatigue and self-reported decline in mood valence can increase over time and may interact with experimental emotion manipulations. Counterbalancing the order of emotion induction administration may help in addressing these concerns. Additionally, these results point to the importance of more continuous assessment of emotion over time, with measures such as psychophysiological monitoring. In particular, recent evidence (Larsen & Norris, 2009; Schaefer et al., 2014) has suggested that deactivation of corrugator muscles (forehead muscles active during frowning), measured through facial electromyography (EMG), may be a highly reliable index of positive emotion valence. Given that experienced valence has been challenging to measure online, this finding provides a promising future methodology for ongoing assessment.

Another important consideration, as previously alluded to, is whether presentation of IAPS pictures on each trial successfully evoked emotion. A passive-viewing run of these images assessed the emotional arousal they elicited via pupil dilation, independent of task-evoked changes in the pupil response. This run followed the general rationale and timing of the protocol used by Bradley et al. (2008), which demonstrated that viewing of emotionally evocative stimuli, relative to neutral, increased pupil dilation and autonomic arousal. Pupil data from the present study did not replicate their findings: Pupil dilation did not change with intended image valence, although pupil dilation differentiated between old and new images, suggesting that pupil responsivity in this run was generally sensitive to relevant experimental factors. Within the AX-CPT, trial-based effects of positive emotion did not lead to lower proactive control / greater reactive control, relative to neutral, although the IAPS images in the present study matched those used by Dreisbach (2006), who successfully demonstrated such a shift. It is important to note, however, that these findings were observed in an experimental design (used by Dreisbach, 2006, and also by Frober & Dreisbach, 2014) that used only trial-by-trial and between-subjects manipulations of positive affect, such that there was no mood induction or intermixing of neutral trials. Additionally, the studies of Dreisbach and colleagues also inserted distractors between cue and probe stimuli. The use of distractors may have increased task difficulty and performance variation, allowing potentially greater sensitivity to observe positive affect influences on behavior. Together, these differences in design may have accounted for some of discrepancies in data patterns.

A related issue is that the valence and arousal associated with the presented IAPS stimuli were based on published norms, but were not acquired from the collected sample (this had also been the case in prior studies using these stimuli with the AX-CPT; e.g., Dreisbach, 2006). Thus, it is unknown whether the specific participants from these studies experienced the presented IAPS stimuli as similarly emotionally evocative. A related possibility, when comparing our passive-viewing results to Bradley et al.’s (2008), is that our run included only positive and neutral images, whereas the Bradley protocol also used negative images. The inclusion of negative images may have created a context in which greater variation in the emotional valence elicited by stimuli is present (Larsen & Norris, 2009), which could amplify the differential response to positive images. Finally, as previously mentioned, the use of a mixed design combining sustained and transient manipulations of positive affect is relatively novel, and it is not yet clear this design can be successful in inducing both tonic and phasic positive affect. Follow-up studies will need to clarify the nature of this design, which may benefit from including a negative valence condition, in addition to positive and neutral, to explore the possibility of emotional context effects.

Although these limitations remain to be addressed by future research, it is important to emphasize that the present study utilized a relatively large sample (*N* = 100) that is, to our knowledge, larger than sample sizes in most other studies in this domain. Our sample size, combined with a within-subjects design, provided strong statistical power to evaluate the effects of the emotion/reward manipulations and directly compare them to each other. Although potential concerns exist regarding the effects of fatigue over time and the emotional validity of the IAPS images used as a trial-by-trial emotion manipulation, self-report evidence indicates that the block-based positive emotion induction did elicit the desired effect. Combined with our large sample size, this provides support that the block-based emotion effects on cognition observed here can be interpreted with relative confidence, despite outstanding concerns about other aspects of the design.

Notwithstanding the study limitations described above, consideration of the contextual (block-based) effects of positive emotion and reward motivation also provide some of the strongest evidence of a qualitative distinction between the two manipulations in terms of their relative influences on cognitive control. First, as was just described, the self-report data suggest that the mood induction was effective in producing a positive affect context during task performance. Second, the observed increase in tonic pupil dilation was equivalent in the positive emotion and reward motivation blocks, which may suggest that the two manipulations had similar influences on arousal. Third, the comparison of the Pos–Neut and Rew–Neut trials provides the tightest experimental contrast between the two conditions, since both trials were preceded by neutral IAPS pictures, received the same performance feedback, and had no reward–performance contingency. Nevertheless, a comparison of these two trial types indicated clear differences in behavioral performance, with the reward motivational context producing a robust increase in behavioral indices of proactive control, whereas the positive-emotion context resulted in a very weak or no effect. As such, the primary implication from the present results is that reward motivation reliably enhances proactive control, whereas positive emotion does not appear to strongly impact either proactive or reactive control.

### Comment on Frober and Dreisbach (2014)

We would also like to briefly discuss the findings of Frober and Dreisbach (2014; hereafter, F/D), who similarly examined effects of positive affect and performance-contingent (and additionally, random) rewards on cognitive control. First, the two studies strongly converge in terms of the high-level pattern of results: reward motivation is associated with enhanced proactive control (i.e., selective increase in AY errors), but clearly dissociates from the effects of positive emotion. However, one apparent difference in the results is that F/D report decreased proactive control under positive affect relative to neutral, a finding that stands in contrast to our observation that proactive control increased slightly under positive affect.

Again, it is important to acknowledge the subtle, but clear, differences in the designs of these two studies. Specifically, F/D, but not our study, involved (a) a distractor AX-CPT variant associated with increased difficulty, which may have increased its susceptibility to positive affect; (b) a fully between-subjects manipulation of emotion/reward; and (c) only trial-by-trial manipulations of positive affect. Additionally, an interesting feature of the designed used by F/D was that it combined manipulations of positive affect and performance-contingency of rewards within an experiment session, allowing interactions between these manipulations to be examined. This design revealed that when positive affect and performance contingency were combined, proactive control increased, as opposed to decreased proactive control under the positive affect manipulation alone.

Together, the two studies converge to suggest that incentive manipulations may be more robust than positive-affect manipulations, at least within this experimental context (i.e., influences on AX-CPT performance). Both sets of data also appear to indicate that positive affect effects may be somewhat fragile, and interact with other experimental factors. Given these possibilities, the mixed results of positive affect on cognition (i.e., the discrepant results between both our and F/D’s studies and the diverging influences of pre- vs. postgoal affect found by Gable & Harmon-Jones, 2011) illustrate the need for further work to clarify why affective manipulations may be weaker than incentive manipulations, and to define boundary conditions for eliciting positive-affect effects one way or another (i.e., task difficulty, the nature of the eliciting stimuli, the role of individual differences, etc.).

### Conclusion and directions for future research

The present study provides one of the first direct experimental comparisons of the effects of reward motivation and positive emotion on cognitive control within a single-sample and using a closely matched design. The results clearly indicate differences in the effects of these two manipulations in terms of both directionality and magnitude, with reward motivation exerting a robust influence on proactive control, whereas positive emotion had much weaker effects. As such, the results indicate that these two constructs are distinct and dissociable in terms of their influences on cognitive control. The present study also highlights the complexity and challenges of inducing positive emotion in the lab in an ecologically valid manner and sustaining it over time in order to examine its effects on cognition. Although we used positive emotion inductions from previous studies (i.e., Dreisbach, 2006; Gray, 2001), our design was not a direct replication of either study’s protocol, combining multiple inductions within a single design as well as incorporating pupillometry data collection. Effects on performance and pupil data departed from previous findings, and self-reported assessments suggested that positive mood was difficult to sustain.

Another key finding emerging from multiple analyses in the present study is that phasic pupil dilation may serve as not just as a coarse indicator of mental effort, but as a more specific indicator of proactive control. This finding contributes to the growing literature highlighting the utility of pupillometry for theoretically driven investigations of distinct cognitive processes (Chatham, Frank, & Munakata, 2009; Chiew & Braver, 2013; Einhäuser, Koch, & Carter, 2010). In contrast, whereas our data indicate important dissociations and interactions between tonic and phasic pupil dilation, the present results diverge from prior theoretical accounts regarding the relationship between the these two components (Gilzenrat et al., 2010). In particular, the presence and equivalence of tonic pupil effects in the emotion and reward conditions, in conjunction with very divergent behavioral effects, suggest that the pupil is also potentially sensitive to affective/motivational factors that are not tightly coupled to cognitive change (e.g., autonomic processes or arousal). Elucidating the neurobiological mechanisms underlying these effects is critical, as pupil dilation has been primarily linked to LC-NE system activity, whereas reward motivation and positive emotion have been primarily associated with DA system influences.

One potentially fruitful direction for future research would be to explore eye-blink rate (EBR) as a potentially complementary measure that may potentially shed light on the relationship between LC-NE and DA activity in relationship to reward and positive affect. Evidence from psychopathology and pharmacological manipulations indicates that spontaneous EBR may serve as a functional marker of central/tonic DA function (Colzato, van Wouwe, & Hommel, 2007; Taylor et al., 1999), may also show sensitivity to positive mood induction (Akbari Chermahini & Hommel, 2012). In addition, a recent study combining data collection of both pupil dilation and blink rate as peripheral measures of NE and DA function during conflict adaptation (van Bochove, Van der Haegen, Notebaert, & Verguts, 2013) revealed that blinks predicted similar conflict adaptation on subsequent trials, providing evidence that a blink index during task performance may predict trial-to-trial performance. Combining pupil dilation and blink measures in a similar manner with the AX-CPT paradigm may offer a powerful approach to clarify the complementary roles of NE and DA during task performance. Unfortunately, calculating blink rate in the present dataset may not be appropriate: We did not collect electrooculographic (EOG) data to index blinks, as Hommel and colleagues did (Akbari Chermahini & Hommel, 2012; Colzato et al., 2007), and unlike van Bochove and colleagues (2013), who assumed that missing data in their pupil dilation measure connoted a blink, we used a headrest supporting the back of the head (instead of a full chin-and-forehead headrest, as van Bochove et al. had used), which was more comfortable for participants but also increases uncertainty that missing data in the pupil stream is a blink, versus due to head movement.

An important additional issue in examining the effect of reward incentives on cognitive performance is exploring the effect of different reward schedules. In the present study, participants were awarded incentives on the basis of both accuracy and speed (via an RT cutoff, following the reward schedule used in Chiew & Braver, 2013). Given that error rates in the present paradigm were relatively low under baseline, this seems to be a less powerful manipulation. However, we would have expected that under such conditions, the RT patterns (which were consistent with the error data) would have been accentuated.

Finally, the role of stimulus novelty in modulating control is an important issue to be addressed by future work. The emotion manipulation in the present study was such that, within the positive block, Pos–Neut images were not novel (i.e., had been previously viewed in the neutral block), but Pos–Pos images were (i.e., had not been previously viewed). Given evidence that novel stimuli may be automatically prioritized relative to familiar stimuli, modulating interference in a Stroop task (Krebs, Fias, Achten, & Boehler, 2013), it may be possible that the positive stimuli viewed in the present study were similarly prioritized on account of being novel rather than on account of their emotional valence, leading to greater proactive control on Pos–Pos trials relative to Pos–Neut trials. Future work fully controlling for novelty when examining emotional valence effects on control will help clarify this issue.

In sum, our results provide converging evidence that reward motivation can be used to robustly and reliably enhance proactive control. In contrast, the effects of positive emotion were much weaker, but if anything, pointed in the same direction. As such, the findings are not consistent with prior reports that such positive-emotion manipulations may reduce and/or enhance proactive control. Further research will be needed, not only to clarify why several hypotheses based on previous evidence were not fulfilled, but more importantly, to more thoroughly characterize the relationship between emotion and motivation and their interaction with ongoing cognitive processing.

## Electronic supplementary material

Below is the link to the electronic supplementary material.

ESM 1 (PNG 45 kb)

ESM 2 (PNG 86 kb)

ESM 3 (PNG 52 kb)

ESM 4 (PNG 51 kb)

ESM 5 (PNG 50 kb)

ESM 6 (PNG 87 kb)

ESM 7 (DOCX 142 kb)

Table S1 (DOCX 77 kb)

## Footnotes

1 The neutrally valenced images used in the AX-CPT were those in files 7000.BMP, 7002.BMP, 7004.BMP, 7006.BMP, 7009.BMP, 7010.BMP, 7020.BMP, 7025.BMP, 7030.BMP, and 7034.BMP. The positively valenced images used in the AX-CPT were from files 1440.BMP, 1463.BMP, 1710.BMP, 2050.BMP, 2057.BMP, 2058.BMP, 2250.BMP, 2311.BMP, 2341.BMP, and 2345.BMP

2 Although the index assumes the complementarity of AY and BX performance, proactive and reactive control dimensions might be somewhat independent in certain contexts. Thus, an important goal for future research will be to explore alternative indices of proactive and reactive control that do not depend on such assumptions.
