# Supplementary figures and images for: Dissociable influences of reward motivation and positive emotion on cognitive control
Source: Cogn Affect Behav Neurosci. 2014 Apr 15;14(2):509–29. doi: 10.3758/s13415-014-0280-0 (PMC4072919; doi:10.3758/s13415-014-0280-0)

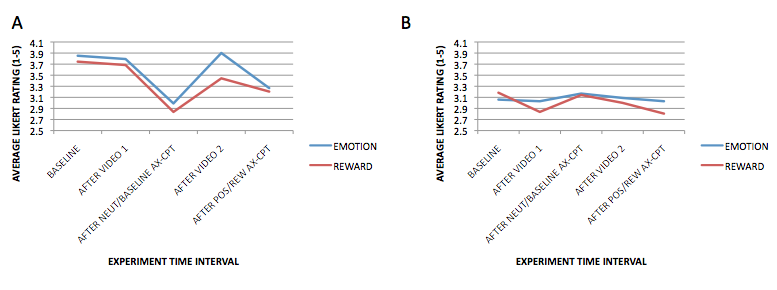

Supplement: Supplementary file 1 — (PNG 45 kb) [file 13415_2014_280_MOESM1_ESM.png]

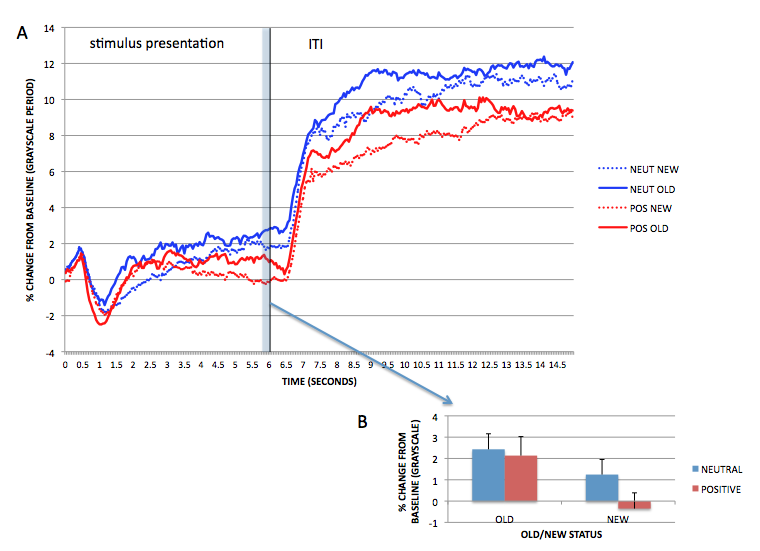

Supplement: Supplementary file 2 — (PNG 86 kb) [file 13415_2014_280_MOESM2_ESM.png]

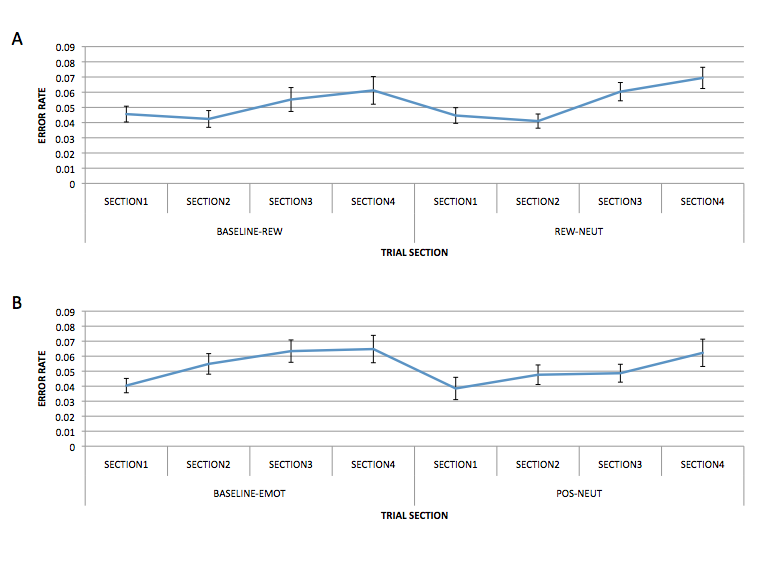

Supplement: Supplementary file 3 — (PNG 52 kb) [file 13415_2014_280_MOESM3_ESM.png]

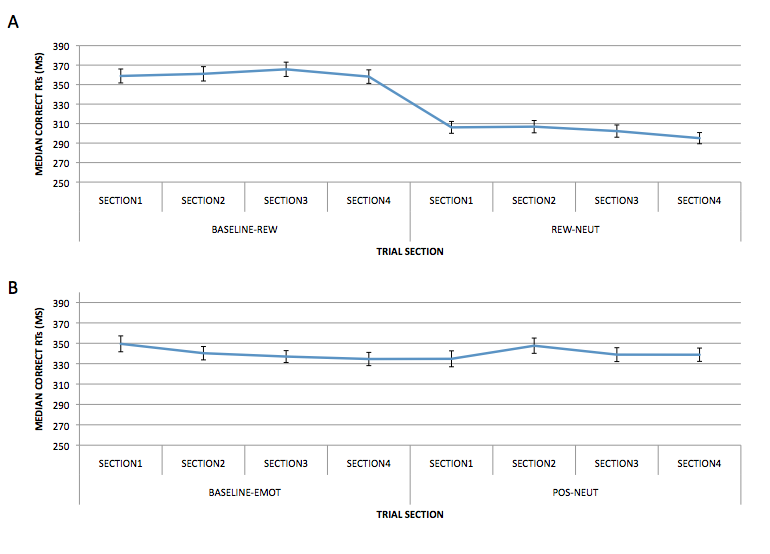

Supplement: Supplementary file 4 — (PNG 51 kb) [file 13415_2014_280_MOESM4_ESM.png]

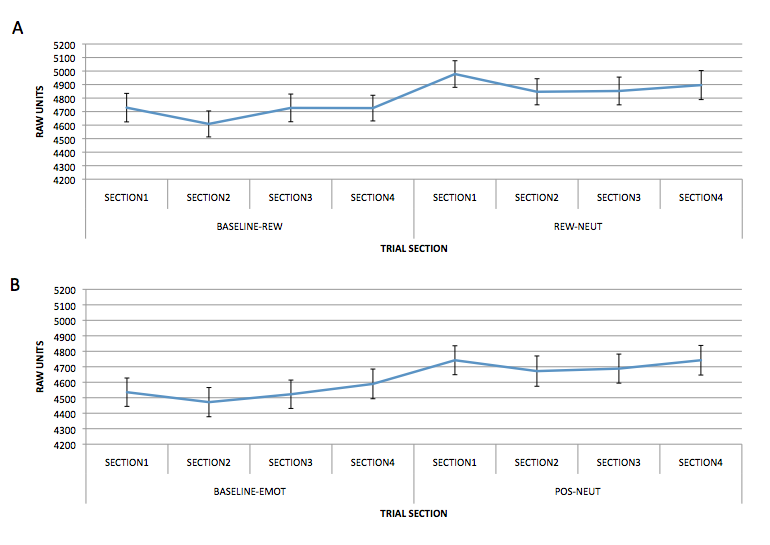

Supplement: Supplementary file 5 — (PNG 50 kb) [file 13415_2014_280_MOESM5_ESM.png]

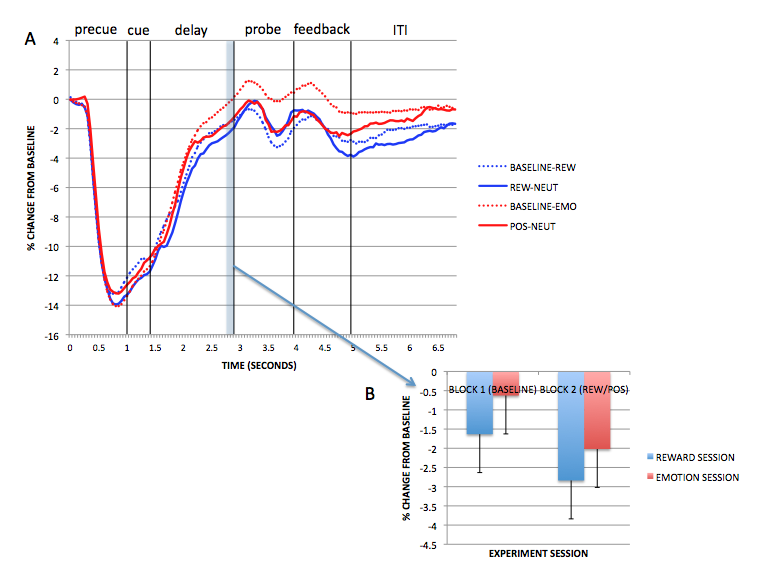

Supplement: Supplementary file 6 — (PNG 87 kb) [file 13415_2014_280_MOESM6_ESM.png]
