# Supplementary material for: Dissociable influences of reward motivation and positive emotion on cognitive control
Source: Cogn Affect Behav Neurosci. 2014 Apr 15;14(2):509–29. doi: 10.3758/s13415-014-0280-0 (PMC4072919; doi:10.3758/s13415-014-0280-0)
Supplement: Supplementary file 7 — (DOCX 142 kb) [file 13415_2014_280_MOESM7_ESM.docx]

Supplementary Methods

*SAM Assessments*

Subjective emotional experience was assessed over the course of the experiment with the Self-Assessment Manikins (SAM; Bradley and Lang, 1994), administered by computer. Participants rated the valence and arousal of their currently experienced emotional state on a 5-choice scale, with “manikins” (non-verbal characters) illustrating the choices. On valence these ranged from 1 (very negative) to 5 (very positive) with 3 (i.e., middle score) being neutral. On arousal, these ranged from 1 (no/very low arousal) to 5 (very high arousal) with 3 being moderate arousal. Participants made five SAM ratings over the course of each experimental session. Thus, in the Reward (Emotion in brackets) session, participants made SAM ratings (1) upon arrival (i.e., at baseline); (2) after watching a ~10 minute neutral video clip; (3) after completing the Neutral (Baseline) AX-CPT block; (4) after watching a ~10 minute neutral (positive) video clip; (5) after completing the Reward (Positive) AX-CPT block.

*Passive Viewing Run of IAPS images*

A passive viewing run of the IAPS followed the AX-CPT task blocks in the Emotion session. This run was included to examine the extent to which pupil dilation increased with arousal while viewing emotional stimuli in a relatively task-free context (following experimental procedure used by Bradley et al., 2008). Half of the images were old (previously seen in the AX-CPT blocks) and half were novel^[[1]](#footnote--1)^. Average normed valence and arousal of the novel images was matched to the images previously viewed during the AX-CPT (normed values in Table S1). All four image types (pos/neu x old/new) were randomly intermixed. Following Bradley et al. (2008), participants viewed each image for 6 seconds, with a 10-second ITI between them. Each picture was preceded by a 2 second grayscale slide (matched to average luminosity of the image set) to control illumination level prior to picture onset.

Participants were told that their memory for the images would be tested immediately after (to ensure that participants remained on task); a brief recognition test consisting of ten images (five presented in the passive viewing run, five completely novel) was administered following the run but was not scored.

Supplementary Results

*Effects of Emotion Inductions: SAM Assessments*

Participants gave 5 SAM ratings during each session (ten in total). Average valence and arousal ratings for participants with complete SAM data (N=91) are shown in Figure S1. Separate repeated-measures ANOVAs tested for effects in valence and arousal, with session and time interval as within-subjects factors and gender and session order (Emotion first and Reward second, or vice versa) as between-subjects factors. For valence, the ANOVA revealed a significant main effect of time interval [*F*(4,356) = 39.450, *p* < .001], a significant main effect of session [*F*(1,89) = 4.224, *p* = .043], a significant session x time interaction [*F*(4,356) = 2.692, *p* = .031], and a significant session x time x order interaction [*F*(4,356) = 2.676, *p* = .032].

The 3-way interaction was due to the fact that rated valence over time was different in the Emotion session as a function of session order [in Emotion session data, time x order interaction was *p* = .05], while in the Reward session, rated valence over time did not differ as a function of session order [time x order interaction, *p* = .206]. Valence in the Emotion session followed similar general patterns in both session orders: participants’ mood valence stayed stable with neutral video viewing, decreased after the Neutral block, rose again with positive video viewing, and decreased again after the Positive block. However, those in the Emotion session first reported larger increases/decreases in mood valence over the Emotion session than individuals who completed the Emotion session second (i.e., following the Reward session) In a focused analysis of valence ratings by time in the Emotion session, the effect of interval was *p* = .006 for Emotion session first subjects, and *p* = .031 for Emotion session second subjects.

These session effects appear to suggest that the positive emotion induction was more effective when participants were engaging in it on their first session visit than when they were returning for their second session. Reward session data followed a similar pattern to Emotion session data (stable mood valence with first neutral video viewing, decreased mood valence after the Baseline block, increased mood valence following the second neutral video). Across both session orders, increased mood valence from interval 3 to 4 (before and after watching the second video) was numerically larger in the Emotion session than in Reward (average increase of 0.97 +/- 0.10, compared to 0.685 +/- 0.12), as should be expected with viewing of a positive video (Emotion session) as opposed to a neutral video (Reward session).

Importantly, when we examined valence ratings specifically from interval 3 to 4 as a function of session (in a separate 2 x 2 ANOVA with interval and session as factors), we observed a significant interval x session interaction [*F*(1,92) = 5.249, *p* = .024]: while mood valence increased from interval 3 to 4 in both sessions, mood valence became significantly more positive after viewing the positive video (i.e., Emotion session) versus a second neutral video (i.e., Reward session). This interaction suggests the positive video increased positive mood as intended, significantly more than a neutral video.

Valence at the interval from 4 to 5 (i.e., before versus after the second task block) decreased in both sessions, but a 2 x 2 ANOVA with session (Emotion, Reward) and interval (interval 4, interval 5) as within-subject factors revealed a significant interaction of session x interval [*F*(1,90) = 4.897, *p* = .029]: this decrease was greater in the Emotion session. In other words, participants reported feeling more positive at interval 4 in the Emotion session (after the positive induction video) than in the Reward session (after a neutral video), but returned to similar mood valences in both sessions at interval 5.

For arousal, the ANOVA revealed a trend-level main effect of time [*F*(4,356) = 1.999, *p* = .094] and a trend-level main effect of order [*F*(1,89) = 3.538, *p* = .063], but no effects of session were significant [*F* < 1 for session main effect and all interactions]. The trend-level order effect was due to higher arousal in participants who completed the Emotion session first. Within-subjects simple contrasts of arousal data at individual time intervals revealed that the trend-level effect of time was due to a decrease in arousal from the first assessment interval (i.e., upon experiment start) to second assessment interval (i.e., after viewing a neutral video clip; *p* = .009) and then an increase again from second to third (i.e., after completion of the first block of the AX-CPT; *p* = .027]. Compared to valence, reported mood arousal stayed more stable over the course of each session; this is consistent with previous studies where greater change in self-reported mood valence than arousal has been observed in response to positive mood induction in the laboratory (Bruyneel et al., 2012). Less is known about the effect of reward incentives on mood; while some research suggests that incentives may promote positive mood (Meloy et al., 2006) and more primary appetitive stimuli (such as erotic pictures) may increase physiological arousal (Walter et al., 2008), to our knowledge no previous studies have explicitly investigated self-reported mood valence and arousal under monetary incentive.

*Effects of Emotion Inductions: IAPS Passive Viewing Run*

In this run, pupil dilation in response to passive viewing of IAPS images was examined as a function of valence (neutral versus positive) and old/new status. There were no overt task performance measures for the passive viewing run, only pupil activity in response to stimulus presentation. Pupil timecourses were extracted as a percentage change measure relative to the last 100ms of grayscale presentation prior to IAPS image onset for each trial and are shown in Figure S2 (full timecourses shown in Figure S2a). Average pupil dilation during the last 250ms of IAPS image presentation (timepoints 5750-6000ms; shown in Figure S2b) was examined using mixed-model analysis. We chose this epoch specifically because it occurred at the end of the image presentation, when the image had already been presented for multiple seconds, in order to compare pupil magnitudes at maximum possible differentiation as a function of valence; given that Bradley et al. (2008) observed differing effects of valence on pupil dilation over a 4-second period (2-6 seconds following image onset), we anticipated that our measurement should permit examination of valence effects with equal or greater strength to their investigation.

Passive viewing data was available for 89 subjects (3255 usable observations in total). Because each IAPS image shown had a different average luminosity that could have influenced pupil diameter, the mixed model analysis statistically controlled for variation in luminance (and its associated effect on pupil dilation) on each trial when examining the two factors of interest (valence and old/new). The model revealed a significant effect of old/new status [*F*(1,3163) = 22.53, p < .001] but no significant effect of valence [*F*(1,3163) = 0.481, *p* = .49] or interaction [*F*(1,3163) = 1.452, *p* = .23]: pupil dilation was greater during viewing of old versus new images, but did not significantly vary with emotional valence. The old/new effect is consistent with previous literature suggesting that pupil dilation is greater when viewing old compared to new items on recognition memory tests (Otero et al., 2011; Vo et al., 2008).

The observed null effect of valence is surprising, given previous reports that pupil dilation is greater when viewing emotionally evocative stimuli compared to neutral stimuli as a result of autonomic arousal (Bradley et al., 2008). While Bradley’s study examined average pupil dilation over the full 4-second viewing period as opposed to our focus on the last 250ms of viewing, visual inspection of our pupil timecourses for the full viewing period (Figure S2) suggest that pupil dynamics as a result of valence remained stable, indicating that examining pupil dilation over the full viewing period would likely yield the same results. Given that pupil dilation during the passive viewing run showed sensitivity to memory effects, the null effect of valence may reflect failure of the positive IAPS images to elicit emotion (or at least emotion-related changes in autonomic arousal), as opposed to poor pupil sensitivity to psychological influences. This possibility is discussed in the main Discussion section.

*Full Analyses of Behavioural Performance During AX-CPT*

In the main text, we provided a focused description of significant effects of critical interest from the analyses of reward and positive emotion influences on AX-CPT performance. Here, we provide a full description of significant effects in these analyses.

*Reward AX-CPT*

Block-based analyses of incentive compared task performance on trials in Baseline-Rew and Rew-Neut trials. The error rate ANOVA revealed a significant main effect of block [*F*(1,99) = 4.806, *p* = .031; Reward > Baseline], a significant main effect of cue [*F*(1, 99) = 60.234, *p* < .001; A > B], a significant main effect of probe [*F*(1,99) = 64.818, *p* < .001; Y > X], a significant interaction of block x cue [*F*(1,99) = 60.444, *p* < .001; A > B effect larger in Reward block than Baseline] a significant interaction of block x probe [*F*(1, 99) = 27.762, *p* < .001; Y > X effect larger in Reward block than Baseline], a significant interaction of cue x probe [*F*(1,99) = 94.356, *p* < .001; AY > all other trial types], and a significant interaction of block x cue x probe [*F*(1,99) = 31.780, *p* < .001; AY error effect greater in Reward than Baseline block]. As described in the main text, these significant effects were due to increases in AY errors in Rew-Neut relative to Baseline-Rew (i.e., the block x cue x probe interaction); significant main effects and two-way interactions were driven by this pattern. The RT ANOVA revealed a significant main effect of block [*F*(1,99) = 123.171, *p* < .001; Baseline > Reward], a significant main effect of cue [*F*(1,99) = 538.871, *p* < .001; A > B], a significant main effect of probe [*F*(1,99) = 829.668, *p* < .001; Y > X], and a significant interaction of cue x probe [*F*(1, 99) = 526.131, *p* < .001; AY > all other trial types]. While RTs were faster overall in the Reward block, this effect did not interact with trial type (which showed expected slowing in AY trials).

Trial-based analyses of incentive compared task performance on Rew-Neut and Rew-Rew trials. The error rate ANOVA revealed a significant main effect of incentive [*F*(1,99) = 18.813, *p* < .001; Rew-Rew > Rew-Neut], a significant main effect of cue [*F*(1,99) = 171.773, *p* < .001; A > B], a significant main effect of probe [*F*(1,99) = 160.113, *p* < .001; Y > X], a significant interaction of incentive x cue [*F*(1,99) = 45.749, *p* < .001; A > B effect larger in Rew-Rew than Rew-Neut trials], a significant interaction of incentive*probe [*F*(1,99) = 52.687, *p* < .001; Y > X effect larger in Rew-Rew than Rew-Neut trials], a significant interaction of cue x probe [*F*(1,99) = 172.720, *p* < .001; AY > all other trial types], and a significant interaction of incentive x cue x probe [*F*(1,99) = 42.047, *p* < .001; AY > all other trial types effect larger in Rew-Rew than Rew-Neut trials]. These significant results are due to a pattern very similar to that under block incentive effects (increased AY errors and decreased errors in all other trials) indicating that transient incentive effects were associated with an additional shift towards proactive control. The RT ANOVA revealed a significant main effect of incentive [*F*(1,99) = 93.942, *p* < .001; Rew-Neut > Rew-Rew], a significant main effect of cue [*F*(1,99) = 684.028, *p* < .001; A > B], a significant main effect of probe [*F*(1,99) = 522.369, *p* < .001; Y > X], and a significant interaction of cue x probe [*F*(1,99) = 516.906, *p* < .001; AY > all other trial types]. These results indicated typical trial-type effects (slower RTs in AY trials than in other trial types) and an overall speeding of RTs with incentive, but no interaction with trial-type.

*Emotion AX-CPT*

Block-based analyses of positive emotion compared task performance on Baseline-Emot and Pos-Neut trials. The error rate ANOVA revealed a significant effect of cue [*F*(1,99) = 29.988, *p* < .001; A > B], a significant effect of probe [*F*(1,99) = 51.635, *p* < .001; Y > X], a significant interaction of block x cue [*F*(1,99) = 10.755, *p* = .001; A > B effect larger in Pos-Neut than Baseline-Emot], a significant interaction of block x probe [*F*(1,99) = 4.927, *p* = .029; Y > X effect larger in Pos-Neut than Baseline-Emot], and a significant interaction of cue x probe [*F*(1,99) = 80.321, *p* < .001; AY > all other trial types]. As described in the main text, these findings indicate a small shift towards proactive control with positive emotion. The RT ANOVA revealed significant main effects of cue [*F*(1,99) = 485.530, *p* < .001; A > B], probe [*F*(1,99) = 550.942, *p* < .001; Y > X], and a significant interaction of cue x probe [*F*(1,99) = 651.708, *p* < .001; AY > all other trial types]. These effects were due to elevated AY RTs relative to all other trial types. No significant emotion effects were observed in this analysis.

Trial-based analyses of positive emotion compared task performance on Pos-Neut and Pos-Pos trials. The error rate ANOVA revealed significant main effects of cue [*F*(1,99) = 46.276, *p* < .001; A > B], probe [*F*(1,99) = 58.853, *p* < .001; Y > X], and a significant interaction of cue x probe [*F*(1,99) = 64.302, *p* < .001; AY > all other trial types]. These results were due to standard task effects (elevated AY errors) but no emotion effects. The RT ANOVA revealed a trend-level main effect of emotion [*F*(1,99) = 3.771, *p* = .055; Pos-Neut > Pos-Pos], a significant main effect of cue [*F*(1,99) = 568.255, *p* < .001; A > B], a significant main effect of probe [*F*(1,99) = 574.267, *p* < .001; Y > X], a significant interaction of emotion x cue [*F*(1,99) = 5.175, *p* = .025; A > B effect larger in Pos-Pos than Pos-Neut], and a significant interaction of cue x probe [*F*(1,99) = 574.268, *p* < .001; AY > all other trial types]. As described in the main text, these results were due to elevated RTs in AY trials and decreased RTs in all other trial types with positive emotion, consistent with increased proactive control.

*Analyses on First Session Data*

While experiment session (Reward and Emotion) was counterbalanced across participants, examination of self-reported emotion (via SAM assessments) suggested that the emotion induction may have been more powerful for participants engaging in it on their first session visit, as opposed to their return visit for the second experimental session. To examine whether session order significantly impacted performance, we re-ran key analyses of performance and pupil activity in data from the first session only (Emotion session first, N=51; Reward session, N=49 with intact performance data) and compared the outcomes to analyses of the full dataset with both session orders.

To provide a focused examination of task performance under emotion and reward manipulations, we analyzed proactive indices (following *Proactive Control Focused Analyses and Comparisons* in the main text). For participants who completed the Reward session first, block and trial-based effects of the reward incentive manipulation were examined on proactive indices using paired-sample *t­-*tests. Block-based incentive effects were associated with increased proactive indices [errors: *t*(48) = 4.325, *p* < .001; RT: 2.649, *p* = .011], while trial-based incentive effects were associated with an increased proactive index for errors [*t*(48) = 3.911, *p* < .001] but not for RTs [*t*(48) = .256, *p* = .799]. This is highly consistent with results from the full dataset, where proactive indices increased on a block-related basis for both errors and RTs (*p* < .001), and on a trial-related basis for errors (*p* < .001) but not RTs (*p* = .625). This suggests that session order did not have much influence on task performance in the Reward session.

Next, we conducted a parallel analysis examining block and trial-related effects of positive emotion on participants who completed the Emotion session first. These revealed a trend-level block-based effect in error proactive indices [*t*(50) = 1.931, *p* = .059; due to higher proactive index in Pos-Neut versus Baseline-Emo trials] but not in RT proactive indices [*t*(50) = .421, *p* = .676]. Trial-based effects were not significant for proactive indices in either measure [errors: *t*(50) = -.373, *p* = .710; RTs: *t*(50) = 1.120, *p* = .268]. In contrast, analysis of the full dataset revealed no significant block-based effects of emotion on proactive indices, but a significant trial-based increase in RT proactive indices (*p* = .013) as previously mentioned. Both analyses are consistent with increased proactive control under positive emotion, but block-based error effects appear to be stronger and trial-based RT effects appear to be weaker in the first session-first participants, possibly reflecting a stronger influence of the video clips (as suggested by the SAM assessments; i.e., a relatively block-based, as opposed to trial-by-trial, influence) in these participants.

We followed up these analyses of proactive index measures with post-hoc simple contrasts examining, in Emotion-session-first participants, block and trial-based emotion effects within measures (error rates and RTs) for each trial-type. Although proactive index analyses suggested that block-based emotion effects (in errors) were stronger in first session data, significance levels of AY and BX analyses did not change for errors (significance changes from full dataset marked with asterisks; AX: *p* = .069; AY: *p* = .322; BX: *p* = .028; BY: *p* = .121*) although first-session AY RTs were actually faster in Pos-Neut than Baseline-Emo conditions (AX: *p* = .074; AY: *p* = .038*; BX: *p* = .087; BY: *p* = .246). Significance levels in the trial-based contrast did not change for errors (AX: *p* = .957; AY: *p* = .850; BX: *p* = .672; BY: *p* = .821) and became weaker in RTs (AX: *p* = .065*; AY: *p* = .719; BX: *p* = .099*; BY: *p* = .827).

To examine the effect of session order on emotion and reward-related changes in pupillometric activity, we conducted two key analyses on first-session data and compared results to the full dataset. Block-based effects of incentive and emotion on pupil activity were examined in separate analyses. Pupil magnitude at the pretrial period (-200-0ms) in first-session data was compared between Baseline-Rew and Reward blocks to examine the block-based incentive effect, and between Baseline-Emo and Positive blocks to examine the block-based emotion effect. The block-based Reward contrast (Reward > Baseline, as in the full dataset) did not reach significance [*t*(42) = 1.100, *p* = 2.78] as it did in the full dataset (*p* = .008); in contrast, the block-based Emotion contrast reached significance [*t*(47) = 2.722, *p* = .009; Positive > Baseline-Emo], consistent with findings in the full dataset.

Trial-based effects of reward incentive were examined on first-session (i.e., Reward session first) pupil activity at the cue maintenance period (2550-2800ms) in an ANOVA with incentive (Rew-Neut, Rew-Rew conditions within the Reward block) and cue (A, B) as within-subject factors. The ANOVA revealed a significant main effect of incentive [*F*(1,48) = 27.357, *p* < .001; Rew-Rew > Rew-Neut], a significant main effect of cue [*F*(1,48) = 15.477, *p* < .001; B > A] and a trend-level interaction between incentive x cue [*F*(1,48) = 3.591, *p* = .064; B > A effect was greater under incentive]. These results are very similar to what was observed in the full dataset, where both main effects and the interaction all reached significance; the fact that the incentive x cue interaction was only trend-level here may be an issue of statistical power given the smaller sample of first-session data. We similarly examined trial-based effects of positive emotion on first-session pupil activity at cue maintenance in an ANOVA with emotion and cue as within-subject factors. The ANOVA revealed a significant cue effect [*F*(1,50) = 7.108, *p* = .01; B > A] but no significant main effect of emotion [*F*(1,50) = .467, *p* = .498] or emotion x cue interaction [*F*(1,50) = .007, *p* = .936]. This pattern is also very similar to findings in the full dataset, where the B > A cue effect was present but no significant emotion effects were observed.

These results suggest that overall, key experimental findings in the Emotion and Reward sessions were highly similar when examined at first session only, compared to the full dataset. SAM assessments suggest that the emotion inductions may have been somewhat more effective when implemented in the first session, and that this was accompanied by greater changes in proactive index under the block-based emotion contrast. Some reward effects diminished in the first-session analysis compared to full dataset analysis, but these patterns may be partially due to reduced power in the smaller sample size for first session, and overall did not alter the directionality of observed effects.

*Time on Task Effects: Task Performance and Pupil Activity*

These analyses were conducted to clarify the extent to which gradual change in performance and pupil activity may have occurred as a result of time on task effects (i.e., practice, fatigue, possible mood dissipation) and to help clarify whether observed block-related changes in performance/pupil could be attributed to such effects as opposed to the intended experimental manipulations. We divided each of the four AX-CPT blocks (Baseline and Reward blocks in the Reward session, and Neutral and Positive blocks in the Emotion session) into four 50-trial sections each and examined task performance (error rates and RTs) and pre-trial pupil magnitude (in the 200 ms prior to trial start) over the course of these sections. To examine effects on performance and pupil specifically as a result of the block-based contrast, we compared Baseline-Rew trials to Rew-Neut trials and Baseline-Emot trials to Pos-Neut. Results are shown in Figures S3-S5.

In the first set of analyses, separate analyses were conducted for the Emotion and Reward sessions. Performance and pupil measures were analyzed within each experimental session, using 2 x 4 ANOVAs that included task block (first block, second block) and trial section (first, second, third, fourth) as within-subjects factors.

In the analyses of Reward session data, using error rates as a dependent measure (Figure S3a), a significant main effect of section [*F*(3,297) = 11.344, *p* < .001] but no significant effect of block [*F*(1,99) = .349, *p* = .556] or block x section interaction [*F*(3,297) = .508, *p* = .677] was found. This pattern was due to higher errors in sections 3 and 4 than in 1 and 2, in both task blocks. The ANOVA of RTs (Figure S4a) revealed a significant main effect of block [*F*(1,99) = 157.575, *p* < .001], with lower RTs in the Reward block than the Baseline block, but no significant effect of section [*F*(1,99) = 1.676, *p* = .172] or block x section interaction [*F*(1,99) = 1.620, *p* = .185]. With pupil magnitude as a dependent measure (Figure S5a), the Reward session ANOVA revealed significant main effects of both block [*F*(1,84) = 20.847, *p* < .001; Reward > Baseline] and section [*F*(3,252) = 6.956, *p* < .001; due to a decrease in pupil diameter from section 1 to 2, followed by recovery], but no significant block x section interaction [*F*(3,252) = 2.025, *p* = .111]. The lack of a block x section interaction in all three dependent measures is noteworthy because it suggests that whatever time-on-task effects (i.e., the section factor) were present (potentially reflecting boredom or fatigue) did not differ in the Reward block compared to Baseline; thus, the Reward effects were unlikely to be due to a selective time-on-task pattern.

These analyses were repeated in the Emotion session. With error rates as a dependent measure (Figure S3b), the Emotion session ANOVA revealed a significant effect of section [*F*(3,300) = 5.977, *p* = .001], due to increasing errors over the trial sections, but no significant effect of block [*F*(1,100) = 1.836, *p* = .178] or block x section interaction [*F*(3,300) = .952, *p* = .416]. While this effect could be due to increasing boredom or fatigue within a task block, the lack of a significant block x section interaction suggests error patterns were similar in both blocks, and that boredom/fatigue did not “carry over” from one block to the next. In the RT ANOVA (Figure S4b), a significant block x section interaction was observed [*F*(3,300) = 3.274, *p* = .021], but no significant effect of block [*F*(1,100) = .047, *p* = .828] or section [*F*(3,300) = 1.505, *p* = .213]. The block x section interaction was due to significant decreases in RTs from section 1 to sections 3 and 4 in the Neutral block, but a differential pattern in the Positive block, with an increase in RTs from section 1 to section 2. This may indicate a practice effect in the Neutral block that was not present in the Positive block. Yet this effect was not strong enough to drive global differences between task blocks, given a null effect in RTs in the main block-based emotion contrast. With pupil magnitude as a dependent measure (Figure S5b), the Emotion session ANOVA revealed a significant main effect of block [*F*(1,84) = 9.903, *p* = .002; Positive > Neutral] and section [*F*(3,252) = 4.051, *p* = .008; decrease in pupil diameter from section 1 to 2, then recovery], but no significant block x section interaction. [*F*(3,252) = .916, *p* = .433]. In general, the Emotion session seemed to follow a similar pattern to the Reward session, with time-on-task effects being independent of block.

To test the hypothesis that the Reward and Emotion sessions were similar in terms of time-on-task effects more directly, we added session (Reward, Emotion) as an additional factor to the ANOVAs (thus, running a 2 x 2 x 4 ANOVA with session, block, and trial section as factors, on error rates, RTs, and pupil dilation). When conducted on error rates, the ANOVA revealed a significant effect of trial section [*F*(3,297) = 12.244, *p* < .001], due to increasing errors over the course of each task block, but no other significant effects. When conducted on RTs, the ANOVA revealed a significant main effect of block [*F*(1,99) = 107.543, *p* < .001], a significant interaction of session x block [*F*(1,99) = 102.432, *p* < .001], and a significant interaction of session x block x section [*F*(3,297) = 3.198, *p* = .024]. These effects were due primarily to the abrupt decrease in RTs from the Baseline to Reward block in the Reward session, which did not occur in the Emotion session. The session x block x section interaction reflected that the block x section interaction was not significant in Reward session data (*p* = .185), while in the Emotion session data, the block x section interaction reached significance (*p* = .021). While RTs sped up in the reward block relative to baseline, patterns of change in RTs over the course of the block were relatively similar in the baseline and reward block. In the Emotion session data, RT dynamics differed with section between the neutral and positive blocks: the significant interaction is driven by a slowing in RTs in the second section of the positive block that was not present in the neutral block. The ANOVA on pupil activity revealed significant main effects of session [*F*(1,69) = 5.821, *p* = .019; Reward > Emotion], block [*F*(1,69) = 28.708, *p* < .001; Reward/Positive > Baseline/Neutral], and section [*F*(3,207) = 6.774, *p* < .001; characterized by a decrease in pupil diameter from section 1 to 2, followed by recovery], but no significant interactions. Together, these results suggest that the Reward and Emotion sessions were similar in time-on-task effects in errors and pupil dilation, but showed some differences in terms of RT patterns, namely in terms of incentive-related speeding that did not occur under positive emotion.

Finally, we conducted analyses to focus on the transition period between task blocks: specifically examining performance and pupil activity in section 4 of the first (i.e., Baseline/Neutral) block, compared to section 1 of the second (i.e., Reward/Positive) block, as a function of session. This analysis was intended to specifically examine whether performance/pupil change at this transition was greater in the Reward session (i.e., from the Baseline block to the Reward block) than in the Emotion session (i.e., from the Neutral to the Positive block). We thus computed a 2 x 2 ANOVA with session (Emotion, Reward) and trial section (last section of first block, first section of second block) as within-subject factors for each dependent measure. With errors as a dependent measure, the ANOVA revealed a significant main effect of trial section [*F*(1,99) = 9.015, *p* = .003; lower errors in the first section of the second block], but no other significant effects. With RTs as a dependent measure, the ANOVA revealed a significant main effect of trial section [*F*(1,99) = 41.411, *p* < .001] and session x block interaction [*F*(1,99) = 35.386, *p* < .001]. The section effect was due to faster RTs at the beginning of the second task block, relative to the end of the first, but the interaction indicates that this effect was driven by a steep drop in RTs from Baseline to Reward block. With pupil magnitude as a dependent measure, the ANOVA revealed a significant main effect of session [*F*(1,75) = 6.294, *p* = .014; Reward > Emotion] and block [*F*(1,75) = 9.844, *p* = .002; Reward/Positive > Baseline/Neutral] but no significant interaction.

Observed pupil activity and RT effects were generally similar to patterns in Chiew and Braver (2013). Error effects were somewhat different from those previously observed: while in that dataset error rates stayed relatively stable over the four 50-trial sections in each task block, in the present data we observed increases in error rates over time in all task blocks, regardless of experimental manipulation. The protocol in the present study was longer and more complex than that used by Chiew and Braver (2013), and fatigue may thus have occurred to a greater extent and influenced performance more than in that previous study. Also, while pupil diameter was higher in the Reward session than the Emotion session overall, the lack of a significant session x block x section interaction suggests that pupil dynamics as a result of time on task did not significantly differ with reward versus emotion. Overall, changes in performance and pupil activity as a function of trial section within each task block appeared consistent with interpretations that block-based effects were due to intended manipulations of emotion and reward, although error rate data appeared to show some time on task effects, possibly due to fatigue.

*Transient Pupil Effects as a Function of Session and Block Context*

As previously described in the main text, analyses of block and trial-based effects on pupil activity utilized raw pupil data from the pretrial period to identify sustained (block-based) effects of emotion and reward incentive, and normalized pupil data during cue maintenance to identify transient (trial-evoked) effects of these influences. Yet it is possible that these sustained and transient effects may interact. Prior evidence suggests that such interactions may occur under incentive (Chiew and Braver, 2013): greater sustained and decreased normalized (i.e., transient) pupil activity was observed in non-incentive trials, relative to baseline trials, suggesting a shift towards greater sustained activity and decreased transient activity with incentive context.

To examine whether such interactions were present in the current data under both emotion and reward manipulations, we conducted an ANOVA examining trial-evoked (normalized) pupil activity at the cue maintenance period as a function of session and block context. Specifically, a 2 x 2 ANOVA (with Session [Emotion/Reward] x Block [First/Second] as factors) was conducted on pupil magnitude in Baseline-Rew and Rew-Neut trials, and Baseline-Emot and Pos-Neut trials. This analysis aimed to identify any transient activation patterns that might occur in the context of a sustained emotion/reward effect. Visual inspection of normalized pupil time courses indicates that *less* trial-evoked dilation was observed in Rew-Neut and Pos-Neut trials, compared to Baseline-Rew and Baseline-Emo trials (see Figure S6a); this was confirmed by the ANOVA examining pupil dilation at cue maintenance [main effect of block: *F*(1,79) = 7.797, *p* = .007; Figure S6b]. This ANOVA also confirmed that pupil dilation was greater overall in the Reward session than in the Emotion session [*F*(1,79) = 7.295, *p* = .009]. No significant interaction of session x block was observed [*F*(1,79) = .321, *p* = .573], indicating that the decreases in normalized activity from the first block to the second were comparable in the Emotion and Reward session.

Together, these observations are consistent with the hypothesis that normalized, transient pupil activity may be inversely correlated with sustained pupil activity. Specifically, the block effect was due to reduced transient pupil dilations in the emotion/motivation blocks (reward/positive) compared to the baseline blocks for each. While the reward block was associated with increased tonic pupil dilation, it also appears that transient dilation decreased, specifically on non-incentive trials. A similar pattern was observed in the Emotion session, where relatively lower tonic activity but higher transient activity was observed in the neutral block, and in contrast, higher tonic activity but lower transient activity was observed in the neutral trials within the positive block.

Mechanisms underlying these similar patterns of dynamic change in pupil activity under both incentive and emotion contexts are unclear, given that diverging behavioural patterns. As previously described, proactive control (measured by proactive indices) significantly increased with block in the Reward session, but not in the Emotion session. It is possible that different mechanisms underlie these patterns, and that the pupil does not directly reflect changes in cognitive effort alone.

Supplementary Figure Captions

*Figure S1.* Average (a) valence and (b) arousal SAM ratings for the Emotion and Reward sessions (five intervals per session, as specified on the X-axis).

*Figure S2.* (a) Pupil timecourses in the IAPS passive viewing run as a function of valence and old/new status. (b) Averaged pupil magnitude during the last 250ms of IAPS stimulus presentation as a function of valence and old/new status.

*Figure S3.* Task performance as a function of 50-trial sections, with error rates as a dependent measure: (a) in Baseline-Rew and Rew-Neut trials; (b) in Baseline-Emot and Pos-Neut trials.

*Figure S4.* Task performance as a function of 50-trial sections, with RTs as a dependent measure: (a) in Baseline-Rew and Rew-Neut trials; (b) in Baseline-Emot and Pos-Neut trials.

*Figure S5.* Pupil activity during the 200ms pretrial period as a function of 50-trial sections: (a) in Baseline-Rew and Rew-Neut trials; (b) in Baseline-Emot and Pos-Neut trials.

*Figure S6.* (a) Normalized pupil trial timecourses as a function of session (Reward, Emotion) and block context (i.e., Baseline-Rew and Rew-Neut trials in the Reward session, and Baseline-Neut and Pos-Neut trials in the Emotion session). (b) Session and block context effects (as averaged pupil magnitudes) at cue maintenance period (2550-2800ms).

Supplementary Tables

*Table S1.* Norms of IAPS images used in IAPS passive viewing run (taken from (Lang et al., 1999). Task Usage status indicates the old/new status of images at the time of the passive viewing run. Normed valence and arousal ratings were collected on a 9-point Likert scale: for valence, this scale went from most negative to most positive, with 5 connoting a neutral valence; for arousal, this scale went from lowest to highest arousal level.

1. The novel IAPS images used in the passive viewing run were as follows: 2840.BMP, 5534.BMP, 7036.BMP, 7160.BMP, 7161.BMP, 7175.BMP, 7217.BMP, 7235.BMP, 7491.BMP, 7950.BMP (neutral valence); 1600.BMP, 1610.BMP, 1920.BMP, 2071.BMP, 2170.BMP, 2209.BMP, 2550.BMP, 2660.BMP, 5831.BMP, 5910.BMP (positive valence). [↑](#footnote-ref--1)
