# Supplementary material for: Dissociable influences of reward motivation and positive emotion on cognitive control
Source: Cogn Affect Behav Neurosci. 2014 Apr 15;14(2):509–29. doi: 10.3758/s13415-014-0280-0 (PMC4072919; doi:10.3758/s13415-014-0280-0)
Supplement: Supplementary file 8 — (DOCX 77 kb) [file 13415_2014_280_MOESM8_ESM.docx]

Table S1.

| **IAPS File Number** | **Intended Valence** | **Task Usage** | **Normed Valence** | **Normed Arousal** | **Luminance Level** |
| --- | --- | --- | --- | --- | --- |
| 1440 | Positive | Old | 8.19 | 4.61 | 139.7 |
| 1463 | Positive | Old | 7.45 | 4.79 | 105.43 |
| 1600 | Positive | New | 7.37 | 4.05 | 102.72 |
| 1610 | Positive | New | 7.69 | 3.98 | 70.18 |
| 1710 | Positive | Old | 8.34 | 5.41 | 116.23 |
| 1920 | Positive | New | 7.90 | 4.27 | 136.18 |
| 2050 | Positive | Old | 8.20 | 4.57 | 38.29 |
| 2057 | Positive | Old | 7.81 | 4.54 | 205.79 |
| 2058 | Positive | Old | 7.91 | 5.09 | 102.58 |
| 2071 | Positive | New | 7.86 | 5.00 | 86.63 |
| 2170 | Positive | New | 7.55 | 4.08 | 33.67 |
| 2209 | Positive | New | 7.64 | 5.59 | 52.98 |
| 2250 | Positive | Old | 6.64 | 4.19 | 176.78 |
| 2311 | Positive | Old | 7.54 | 4.42 | 121.22 |
| 2341 | Positive | Old | 7.38 | 4.11 | 75.71 |
| 2345 | Positive | Old | 7.41 | 5.42 | 126 |
| 2550 | Positive | New | 7.77 | 4.68 | 106.83 |
| 2660 | Positive | New | 7.75 | 4.44 | 118.82 |
| 2840 | Neutral | New | 4.91 | 2.43 | 87.07 |
| 5534 | Neutral | New | 4.84 | 3.14 | 104.28 |
| 5831 | Positive | New | 7.63 | 4.43 | 139.21 |
| 5910 | Positive | New | 7.80 | 5.59 | 34.24 |
| 7000 | Neutral | Old | 5.00 | 2.42 | 80.22 |
| 7002 | Neutral | Old | 4.97 | 3.16 | 114.51 |
| 7004 | Neutral | Old | 5.04 | 2.00 | 70.89 |
| 7006 | Neutral | Old | 4.88 | 2.33 | 72.08 |
| 7009 | Neutral | Old | 4.93 | 3.01 | 80.16 |
| 7010 | Neutral | Old | 4.94 | 1.76 | 54.16 |
| 7020 | Neutral | Old | 4.97 | 2.17 | 121.69 |
| 7025 | Neutral | Old | 4.63 | 2.71 | 109.78 |
| 7030 | Neutral | Old | 4.69 | 2.99 | 103.08 |
| 7034 | Neutral | Old | 4.95 | 3.06 | 151.98 |
| 7036 | Neutral | New | 4.88 | 3.32 | 102.1 |
| 7160 | Neutral | New | 5.02 | 3.07 | 108.46 |
| 7161 | Neutral | New | 4.98 | 2.98 | 120.65 |
| 7175 | Neutral | New | 4.87 | 1.72 | 48.06 |
| 7217 | Neutral | New | 4.82 | 2.43 | 90.99 |
| 7235 | Neutral | New | 4.96 | 2.83 | 66.17 |
| 7491 | Neutral | New | 4.82 | 2.39 | 39.65 |
